# Extract of *Mallotus oppositifolius* (Geiseler) Müll. Arg. increased prefrontal cortex dendritic spine density and serotonin and attenuated *para-*chlorophenylalanine-aggravated aggressive and depressive behaviors in mice

**DOI:** 10.3389/fphar.2022.962549

**Published:** 2022-10-28

**Authors:** Kennedy K.E. Kukuia, Frimpong Appiah, George J. Dugbartey, Yaw F. Takyi, Patrick Amoateng, Seth K. Amponsah, Ofosua Adi-Dako, Awo E. Koomson, Frederick Ayertey, Kevin K. Adutwum-Ofosu

**Affiliations:** ^1^ Department of Medical Pharmacology, University of Ghana Medical School, College of Health Sciences, University of Ghana, Accra, Ghana; ^2^ Department of Community Health and Medicine, School of Food and Health Sciences, Anglican University College of Technology, Nkoranza, Ghana; ^3^ Department of Pharmacology and Toxicology, School of Pharmacy, College of Health Sciences, University of Ghana, Accra, Ghana; ^4^ Department of Pharmaceutics and Microbiology, School of Pharmacy, College of Health Sciences, University of Ghana, Accra, Ghana; ^5^ Department of Phytochemistry, Center for Plant Medicine Research, Mampong-Akuapem, Ghana; ^6^ Department of Anatomy, University of Ghana Medical School, College of Health Sciences, University of Ghana, Accra, Ghana

**Keywords:** *Mallotus* oppositifolius, serotoninergic system, pCPA-induced aggression, resident-intruder, dendritic spine density, prefrontal cortex

## Abstract

**Background/Aim:** Depression-related aggression is linked to serotonin (5-HT) and dendritic spine alterations. Although *Mallotus oppositifolius* extract (MOE) has potential for reducing this effect, its specific role remains uncertain. Herein, we evaluated this potential and associated alterations in the brain.

**Methods:** A standard resident-intruder model of *para*-chlorophenylalanine (*p*CPA)-induced depression-associated aggression in male ICR mice was used. The resident mice received *p*CPA (300 mg/kg, i. p.) for 3 consecutive days while saline-treated mice served as negative control. The *p*CPA aggressive mice were subsequently treated orally with either MOE (30, 100, 300 mg/kg), fluoxetine (20 mg/kg), tryptophan (20 mg/kg) or saline (untreated *p*CPA group) for 28 days. Locomotor activity was assessed using open field test. Serotonin (5-HT) levels in mice brain and phytochemical fingerprint of MOE were determined by high performance liquid chromatography (HPLC) while gas chromatography-mass spectrometry (GC-MS) was used to identify constituents of MOE. Dendritic spine density and morphology were evaluated using Golgi-Cox staining technique and analyzed with ImageJ and Reconstruct software.

**Results:** Administration of *p*CPA induced aggressive behavior in mice, evidenced by increased attack behaviors (increased number and duration of attacks), which positively correlated with squeaking and tail rattling. MOE treatment significantly reduced these characteristics of aggression in comparison with vehicle (non-aggressive) and untreated *p*CPA groups (*p* < 0.001), and also reduced social exploration behavior. Although the behavioral effects of MOE were comparable to those of fluoxetine and tryptophan, these effects were quicker compared to fluoxetine and tryptophan. Additionally, MOE also markedly increased 5-HT concentration and dendritic spine density in the prefrontal cortex relative to vehicle and untreated *p*CPA groups (*p* < 0.05). Interestingly, these behavioral effects were produced without compromising locomotor activity. GC-MS analysis of the MOE identified 17 known compounds from different chemical classes with anti-inflammatory, antioxidant, neuroprotective and antidepressant activities, which may have contributed to its anti-aggressive effect.

**Conclusion:** MOE decreased depression-associated aggressive behavior in mice *via* increased 5-HT concentration and dendritic spine density in the prefrontal cortex. The MOE-mediated effects were faster than those of fluoxetine and tryptophan. Our finding suggests that MOE may have clinical promise in decreasing aggressive and depressive behaviors.

## Graphical Abstract

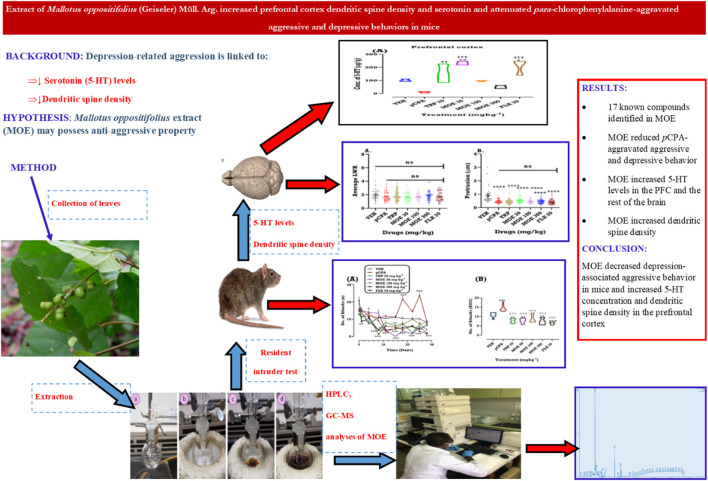

## Highlights

1. Gas chromatography mass spectrometry analysis of the methanol leaf extract of *Mallotus oppositifolius* (MOE) identified 17 known compounds, some of which have anti-inflammatory, antioxidant, neuroprotective and antidepressant activities.
2. MOE produced a faster reduction in para-chlorophenylalanine-aggravated aggressive and depressive behavior when compared to fluoxetine and tryptophan.
3. The anti-aggressive and antidepressant-like effects of MOE were associated with increased prefrontal cortex dendritic spine density and serotonin concentration.
4. The study provides experimental evidence of a plant (*Mallotus oppositifolius*) with phyto-constituents that may have clinical potential for in reducing both depressive and aggressive symptoms.

## Introduction

Depression is a chronic debilitating condition that induces aggressive and suicidal behaviors (Zubrick et al., 2017). It contributes to about 800,000 suicide deaths per year (WHO, 2017). Patients with major depressive disorder have been reported to exhibit enhanced externally directed aggression, reactive aggression and irritability compared to non-depressive subjects (Fritz et al., 2020). Aggression is becoming a major public health issue globally due to the physical injuries and long-term emotional damage on its victims (Siegel and Victorroff, 2009). Aggression is an endophenotype of depression that can be easily modeled and manipulated in animals using the resident-intruder paradigm (Brent, 2009; Studer et al., 2015). Aside the behavioral phenotypes, the neurobiology of depressive and aggressive behaviors can be traced to aberration in the serotonin (5-hydroxytryptamine; 5-HT) system arising from down-regulation of 5-HT function. For example, reduced 5-HT levels in the structures of the forebrain are associated with depression, aggression and suicide (Coccaro et al., 2015; Çetin et al., 2017). Moreover, preclinical studies have demonstrated a correlation between lower levels of 5-HT and its metabolite, 5-hydroxyindoleacetic acid (5-HIAA), and aggression (Kulikov et al., 2012; Wang et al., 2019). Additionally, reduced binding potential of 5-HT_2A_ receptors in the prefrontal cortex (PFC) are associated with aggression and suicide (Mann et al., 2019). Moreover, the essential amino acid tryptophan, the precursor for serotonin synthesis, plays a pivotal role in depression and suicide/aggression, as there are reports showing that tryptophan treatment increased brain serotonin concentration and improved neuronal plasticity in animals (Walz et al., 2013; Alagappan et al., 2020). These pieces of experimental evidence support the assertion that dysregulation of 5-HT neurotransmission and neuronal densities may promote aggressive behaviors. Another neurotransmitter system that is implicated in depression is the glutamatergic system. This assertion is supported by the discovery that ketamine, a glutamatergic antagonist, exhibits both antidepressant and anti-suicide activities through its antagonistic effect on NMDA receptor complex (Kadriu et al., 2019), as well as enhancement of brain-derived neurotrophic factor pathway and opioid pathway (Hosanagar et al., 2021). In addition to the aforementioned, morphological changes, neuronal abnormalities and decreased size and density of neurons in the PFC have been found in depression and aggression (Underwood et al., 2012).

It was previously thought that since depression increases the risk for aggression and/or suicide, antidepressants should reduce aggressive or suicidal behaviors (Hawton et al., 2013; Klonsky et al., 2016). However, there has been a growing controversy in recent years about whether antidepressants increase or decrease the risk aggressive behavior (Stone et al., 2009; Stübner et al., 2018). Most antidepressants seem to improve the energy levels of patients before they improve their mood. This lag in clinical improvement has been shown to increase the risk of aggressive and/or suicide during the early period of treatment (Hammad et al., 2006). This has necessitated the urgent need for antidepressants with aggression-reducing potential. In this regard, plants such as *Mallotus oppositifolius* (Geiseler) Müll. Arg could be an essential source of antidepressants with aggression-reducing potential. Traditional uses of *M. oppositifolius* include epilepsy, depression, pain, inflammation, and infection (Burkill, 1994). A substantial body of empirical evidence, including ours, has shown the therapeutic properties of *M. oppositifolius* extract (MOE) to include CNS depressant, anti-inflammatory, anti-protozoal (anti-blastocystis) and anticonvulsant properties (Kukuia et al., 2012; Nwaehujor et al., 2014; Christensen et al., 2015; Kukuia et al., 2016a; Igwe et al., 2016). We previously reported that mice treated with MOE exhibited a more rapid-onset and sustained antidepressant-like effect *via* enhanced serotoninergic neurotransmission and inhibition of glycine/NMDA receptor complex activation compared to fluoxetine- and imipramine-treated mice (Kukuia et al., 2014; Kukuia et al., 2016b). Based on the above findings, we hypothesized that MOE will reduce depression and associated effects such as aggression. This study examines whether MOE reduces depression and aggressive behavior in mice, as well as its impact on regional brain serotonin levels and dendritic spine morphology and density.

## Materials and methods

### Ethical statement

The experimental protocol (with protocol number CHS-Et/M2-4.9/2019–2020) was approved by the Ethical and Protocol Review Committee, College of Health Sciences, University of Ghana, Korle-Bu. All animals used in this study were handled according to the Guide for the Care and Use of Laboratory Animals (National Research Council, 1996). As part of the 3R principle, all efforts were made to ensure refinement in the conduct of the experiments.

### Chemicals and reagents

*Para*-chloro-phenylalanine (*p*CPA), tryptophan and fluoxetine hydrochloride were purchased from Sigma-Aldrich Inc. St. Louis, Missouri, United States. Potassium dichromate (K_2_Cr_2_O₇), Mercuric chloride (HgCl_2_) and potassium chromate (K_2_CrO₄) were purchased from Merck KGaA, Darmstadt, Germany.

### Plant collection and extraction

Leaves of the *M. oppositifolius* (Geiseler) Müll. Arg (Family: Euphorbiaceae) plant were collected from the Centre for Plant Medicine Research (CPMR), Mampong-Akuapem, Ghana (5°55′05.6″N, 0°08′04.9″W), and authenticated at the same Centre (Voucher Specimen Number: CPMR 4977). The leaves were collected in a manner that did not inflict any harm on the plant. The leaves were air-dried for 7 days after which they were pulverized with a hammer mill into fine powder. Cold maceration method with absolute methanol was used for the extraction for 3 days to mimic the traditional way the plant is used, suggesting that its active ingredient might be in the polar medium. The drug extract solvent ratio was 600 g of the powdered leaves in 6000 ml of the methanol solvent. The resulting extract was concentrated at 60°C and pressure to a syrupy mass on a rotary evaporator. The syrupy mass was dried to a dark brown semisolid mass using water bath and kept in a desiccator to be used later. The final yield was referred to as *M. oppositifolius* extract (MOE).

### Compounds and drugs preparation

The *M. oppositifolius* extract (MOE) was dissolved in distilled *water* and doses of 30, 100 and 300 mg/kg of not more than 0.5 ml per animal were administered orally with the aid of oral gavage. The doses of MOE used in the study were selected on the basis of results obtained from our previous investigation (Kukuia et al., 2012), which stated that neuroactive effects were observed at those doses and that the oral lethal dose LD₅₀ to be above 6000 mg/kg. Appropriate concentrations of tryptophan and fluoxetine were prepared by dissolving them in distilled water and *para*-chloro-phenylalanine (*p*CPA) was dissolved in 2% tween 80 before the start of the experiment. Fluoxetine, a selective serotonin reuptake inhibitor was used as the reference drug, and tryptophan, a serotonin precursor, was used as the positive control and normal saline 0.9% used as the negative control.

### HPLC analysis of plant extract (fingerprinting)

High performance liquid chromatography (HPLC) was used for general profiling of the crude extract based on their adsorption and partition. Fingerprints of the MOE was conducted to separate components of the extracts. Methanol extract of the leaves was analyzed for HPLC fingerprints using Agilent 1100 HPLC apparatus equipped with UV detector (Agilent technologies Santa. Chara CA, United States). HPLC stationary phase used was a TSKgel C18 column (150 mm × 4.6 mm, diameter 3 µm) while the mobile phase was 0.1% phosphoric acid—methanol (95%:5%) with flow rate of 0.7 ml/min, injection volume 20 µl, and the runtime was set at 25 min. The emission wavelength of 300 nm was used.

### Gas chromatography mass spectrometry analysis of the plant extract

Gas chromatography mass spectrometry (GC-MS) analysis of the MOE was performed using a gas chromatograph (PerkinElmer GC Clarus 580) interfaced to a PerkinElmer mass spectrometer (Clarus SQ 8 S) equipped with Elite-5MS (5% diphenyl/95% dimethyl poly siloxane) fused capillary column (30.0 m × 0.25 mm ID × 0.25 μm DF). This required oven temperature initially maintained at 80°C for 1 min, and then increased to 250°C at a rate of 10°C/min and finally to 280°C at a rate of 5°C/min for 10 min. For mass spectrometer detection, an electron ionization system was operated in the electron impact mode. Helium was used as a carrier gas at a constant flow rate of 1 ml/min, and an injection volume of 1 μl was employed. The injector temperature was maintained at 250°C, and the ion-source temperature was kept at 220°C. Mass spectra were taken at 70 eV and a scan interval of 0.5 s over a mass range of 50–450 Da. The time of solvent delay was 0–4 min, and the total GC-MS run time was 40 min. Active components in the MOE were identified by comparison of their retention indices, peak area percentage and mass spectra fragmentation pattern with the mass spectra in the database of National Institute Standard and Technology (NIST) and published literature of spectral data whenever possible. The components from MOE were identified by using similarity indices obtained from Wiley and NIST libraries for GC-MS system used and some published literature of spectral data (Stein, 1990; McLafferty, 2000; Igwe et al., 2016). The relative percentages of the various constituents were expressed as percentages calculated by normalization of the peak area.

### Experimental animals and housing

A total of 119 male Institute of Cancer Research (ICR) mice (20–30 g, at 6 weeks) were obtained from the Noguchi Memorial Institute for Medical Research, University of Ghana and kept at the Animal Experimentation Unit of the Department of Microbiology, University of Ghana Medical School, University of Ghana. Depression and aggression are behavioral states that cannot be modelled *in vitro*, thus, animals were used. The animals were randomly assigned to residents and intruders (49 as residents and 70 as intruders). Each resident was housed in clear stainless-steel cages (28 cm × 17 cm × 14 cm) and intruders maintained in groups of ten, in standard stainless-steel cages (34 cm × 47 cm × 18 cm). The intruders were kept in groups because isolation may induce aggression in them making it difficult to study aggressive behavior in the resident mice. The resident mice were not kept in groups as they may attack themselves after aggression induction with *p*CPA administration. The resident mice were housed in individual cages for 7 days before the experiment with no change in bedding to allow for formation of territorial behavior. An additional 28 days of treatment was permitted while the resident mice were kept in individual cages. The cages were kept with soft wood shavings as bedding materials and the animals fed with normal commercial pellet diet (GAFCO, Tema) and water was made available *ad libitum*. Animals were maintained under required laboratory conditions at ambient temperature of 25 ± 2°C, relative humidity of 60–70%, and under a light: dark cycle of 12:12 h. The animals were acclimatized for 2 weeks prior to the commencement of the experiment. All behavioral studies took place at the Neuropsychopharmacology Research Laboratory, Department of Medical Pharmacology, University of Ghana Medical School, Korle-Bu, Ghana. Aggressive behavior was studied in the dark phase while other behavioral studies were conducted in the light cycle with experimentally naive mice.

### *p*CPA-induced depression-related aggression in mice

A standard resident-intruder model of *para*-chlorophenylalanine (*p*CPA)-induced aggression in 119 male ICR mice (20–30 g) (49 as residents and 70 as intruders) was used for aggression studies as previously described (Studer et al., 2015), with some modifications. Resident mice were randomly divided into 7 groups (n=7), housed in single separate cages and intruders socially housed in 10 per cage. Six out of the 7 groups of the resident mice (Group 1–6) received daily intraperitoneal administration of *p*CPA (300 mg/kg) for three consecutive days to induce aggression and depressive symptoms. The last group received saline and served as non-aggressive control. The resident male ICR mice were screened for aggression using the standard resident intruder test to identify aggressive behavior. Briefly, the mice were tested during the dark phase in their home cage against a naïve intruder mouse for 10 min. The resident male mice were assessed for aggressive behavior toward specific intruder animals housed in social groups throughout the experiment. The intruder mice were weighed and assessed before each behavioral test to ensure that they were of similar weight, age and sex with the resident, while using each intruder mouse once daily (Koolhaas et al., 2013). To distinguish the intruder mice, they were marked on the tail with dye. A single intruder mouse was lightly placed into the home cage in the opposite corner relative to the resident mouse 60 min after oral drug administration and 30 min after intraperitoneal injection. Bites were counted for 5 min following the initial attack. If no attack bite occurred, the session was terminated at 5 min. The resident which did not attack the intruder during this time was considered as non-aggressive. In rare cases where the intruder bit the resident, the intruder was removed immediately and replaced with another group-housed male. After completion of the test, the intruder male was removed from the cage and shavings replaced to eliminate scent cues.

#### Evaluation and scoring of aggressive behavior

Recordings were scored blindly at low video speed by a trained observer blinded to the status of the mice and the experimental procedures. Aggressive behaviors were tracked and recorded as social exploration (including pursuit), threat behaviors (including tail rattling and squeaking) and attack behaviors (including attack frequency and duration). Resident mice that did not display any episode of attack or threat were characterized as non-aggressive. Also, attack latency was set to 300 s if no attack was detected during the entire duration of the test. The tests were videotaped with an overhead light sensitive video camera (Sony 4K Handy Cam, FDR-A×100E) under illumination. All behavioral patterns were evaluated with a computer-based free, versatile open-source event-logging software (Boris v 7.9.6—2019; Friard and Gamba, 2016).

### Drug/extract treatment to evaluate aggression reduction potential

Treatment began the next day after the 3-day *p*CPA-induced aggression as the animals remained isolated in the various treatment groups. Animals in Groups 1–3 received oral administration of MOE at doses of 30, 100, 300 mg/kg respectively while group 4–6 received oral doses of fluoxetine (20 mg/kg), tryptophan (20 mg/kg) and saline respectively. Another group of mice representing group 7 which was not pretreated with *p*CPA also received saline to represent the non-aggressive animals. The experiment lasted for 28 days. The resident-intruder test was used to re-test for aggression at 60 min after drug administration, twice per week with a minimum interval of 72 h for the period of 28 days. The animals were sacrificed after the behavioral studies for histology and biochemical (brain 5-HT concentration) analyses.

#### Forced swimming test

Depression-like behavior in the resident mice were assessed using the forced swimming test (FST) as described by Porsolt et al. (2001) with modifications. This was done to establish a link between aggressive and depressive behaviors. FST was done after the last resident-intruder test specifically, on the 28th day of treatment. Each mouse was gently placed into transparent cylinder (35 cm height, 20 cm internal diameter) filled with water (25°C–28°C) at 15 ± 1 cm height for 6 min. Each session was digitally recorded with a video camera (Sony 4K Handy Cam, FDR-A×100E) suspended approximately 100 cm above the cylinders. An observer scored depression-like behavior for the last 5 min measured as immobility (mice were judged to be immobile when they remained floating passively in the water). Duration of immobility, swimming, and climbing was measured and analyzed using a video tracking software (Boris v 7.9.6—2019).

#### Tail suspension test

Tail suspension test (TST) was conducted as described by Steru et al. (1985) with modifications to test for depression-related behavior in resident rodents. The TST was done after the last resident-intruder test, specifically on the 28th day of treatment. The resident mice were individually suspended by the tail at the edge of a lever suspended above the table top (the distance to the table surface was approximately 35 cm), fixed with adhesive tape located approximately 1–2 cm from the tip of the tail. Opaque cardboard divider was placed between them to block the animals’ view of each other. The test lasted 6 min and the measurements for all three parameters (immobility, swinging, curling) were evaluated in the last 5 min using a video tracking software (Boris v 7.9.6—2019). Depressive behavior was rated as immobility (a mouse was judged to be immobile when it hung by its tail without engaging in any active behavior).

#### Open-field test for locomotor activity

The effect of treatments on locomotor activity of mice was evaluated using the open-field test (Kraeuter et al., 2019a). The resident mice were placed in an open field box with an enclosed floor and walls, and was cubic (60 cm × 60 cm × 25 cm) with an opened top. The center region of the box floor was defined offline as a 20 × 20 cm^2^ but was not marked. Each box was placed on the floor of the experiment room and was dimly illuminated. Each mouse was gently placed in the center of the box and allowed to walk about freely. Locomotor activity was scored as total distance travelled (marked by number of lines crossed) for the 6-min period using a video tracking software (Boris v 7.9.6—2019).

#### HPLC analysis for brain serotonin (5-HT) concentration

The levels of 5-HT neurotransmitter in the resident male mice brain were determined using high performance liquid chromatography (HPLC). Mice were euthanized with diethylether (not more than 5 ml *via* the respiratory route by exposing them to ether for approximately 2 min in a transparent jar) and brain carefully removed from the skull of mouse according to the guidelines set by American Veterinary Medical Association, (2013). Brain samples from both hemispheres were dissected on ice to isolate the prefrontal cortex (PFC) from a thick section in a cryostat. Both PFC and the remaining part of the brain were kept in tissue containers at -80°C until use. Frozen brain samples of mice were weighed in 15 ml falcon tube using electronic balance. The brain tissues were homogenized on ice in 700 µl of ice-cold 0.1% formic acid solution in methanol (1:1, v/v) for 60 s at 60 Hz 30 pulses using a micro-ultrasonic cell disrupter at 30 amplitudes. Brain homogenates were kept on ice for 15 min to aid the precipitation of proteins and cell debris. The homogenates were centrifuged at 14,000 rpm for 20 min at 4°C and the supernatant was collected into a labelled 1.5 ml reaction tube to measure 5-HT in the next section.

HPLC analysis of 5-HT in the supernatant from the brain homogenate was performed on a Shimadzu Prominence reverse phase HPLC consisting of a binary solvent delivery system (LC—20AB), a degasser (DGU-20A3), an auto-sampler (SIL—20ACHT), a column temperature controller (CTO—10AS VP) and a photo diode array detector (SPD—M20A). HPLC stationary phase used was a TSK gel C18 column (diameter 3 μm, length × width, 150 mm × 4.6 mm) while the mobile phase comprised 0.05% formic acid: methanol (90%:10%, v/v) at a flow rate of 0.70 ml/min. The injection volume was 20 µl and the runtime was set at 15 min. The column was heated to 30°C and 5-HT was detected at an emission wavelength of 280 nm. Peaks were identified by comparing their retention time in the sample (tissue extracts) solution with that of standard solution. LC workstation (Shimadzu) software was used to control the HPLC components and to process data.

### Neurohistological studies

Following removal of the brains from the skulls, they were injected with Golgi-Cox staining solution, and processed using a previously described method by Kukuia et al. (2022). Briefly, the injected brains were serially sectioned at a thickness of 100 µm. Every third cryostat section (intervals of 300 µm) was mounted on gelatin-coated sides, stained, dehydrated in ethanol, cleared in xylene, and cover-slipped in Permount (Das et al., 2013). The tissues were processed and spine density assessed by viewing under a light microscope (Zeiss Primo Star with iLED DIN EN 61010–1) at low (×40) and high (×100, with oil emulsion) magnifications. Images were captured using a coupled device eye piece (Lenovo Q350 USB PC Camera) attached to the microscope. The microscope stage was moved around the tissue at 2 and 3 microscope stage unit intervals on the x- and y-axes respectively. Snapshots of the neurons within the field of view were captured (×100) onto a computer (HP Compaq dx2300 Microtower) with the eyepiece. The brain region of interest was the PFC and the dendritic spine density in the PFC were analyzed using ImageJ Software (Fiji version of 1.53c).

### Dendritic spine density

The light microscope with a 100× oil immersion was used to image the sections of labeled neurons and scanned at 1 µm intervals. The dendrite within the frame was cropped (75–200 µm range) according to the extent of dendrite that was clearly connected to the soma of interest and separable from crossing dendrites. Evaluation of dendritic length and spine characteristics was done as previously described (Risher et al., 2014; Zhu et al., 2018). Dendritic spines were analyzed and counted with ImageJ software (Fiji version 1.53c, NIH) and the freely available Reconstruct software (http://synapses.clm.utexas.ed). Further analysis of the spine morphological characteristics was done as described by Risher et al. (2014). Briefly, three independent coronal sections per mouse were used for analysis. A Z-stack images of the Golgi spines were processed with ImageJ software (Fiji version 1.53c, NIH) and imported as series images in the Reconstruct software. The series images were calibrated and processed for the analysis of dendritic spine density and classification. The dendritic segment was identified for all section to be 10 µm in length uninterrupted. The length and width of the spines were measured using the reconstruct software. The data for the spine length and width were imported and computed in a spreadsheet. Spine density was calculated by quantifying the number of spines per dendritic segment, and normalized to 10 μm of dendrite length (Risher et al., 2014; Rosa et al., 2021). The morphological characteristics of the spines were classified as mushroom, filopodia, stubby, long thin, thin and branched, based on the length, width and length-to-width ratio of the spines as described by Risher et al. (2014). The results were then exported to GraphPad Prism for statistical analysis.

### Statistical analysis

GraphPad Prism for windows version 8.0.2 (GraphPad Software, San Diego, CA, United States) was used for all data and statistical analysis. Values were presented as mean ± SEM. *p*-value less than 0.05 between groups was considered statistically significant. Differences in means were analyzed and evaluated by both one-way analysis of variance (ANOVA) and two-way ANOVA test followed by Tukey’s and Bonferroni’s post-hoc test respectively.

## Results

### Effect of MOE on aggressive behavior

**FIGURE 1:**
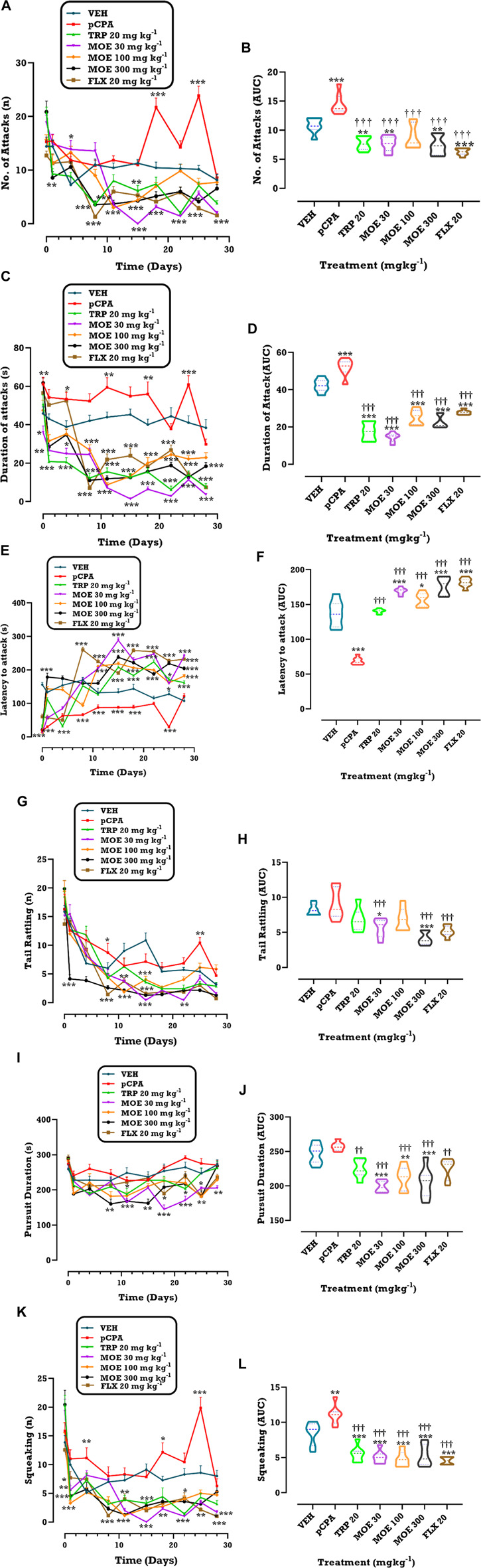
(Continued).

As part of our research questions, we wanted to know what effect MOE will have on aggressive behavior. The results of 28-day treatment with MOE, fluoxetine and tryptophan on *p*CPA-aggravated aggression in the resident-intruder test, measured as attack latency, number of attacks, total duration of attacks, tail rattling, pursuit duration and squeaking are shown in Figures 1A–F. The graphs are presented as time course curves (Figures 1A,C,E,G,I,K) and violin plots (as AUCs; Figures 1B,D,F,H,J, 1L). All treatments (MOE, fluoxetine and tryptophan) significantly reduced the total number of attacks (F₆,₄₂=25.65, *p* < 0.0001) (Figure 1A) and total duration of attacks (F₆,₄₂=100.6, *p* < 0.0001) (Figure 1C) compared to untreated *p*CPA group. Interestingly, MOE and tryptophan treatments reduced the attack behavior from day 1 compared to fluoxetine, which started on day 8. Also, the graphs (Figures 1B,D) showing the areas under the curve for the treatment groups (MOE, fluoxetine, tryptophan) showed significant reduction in total number of attacks (F₆,₄₂=25.65, *p* < 0.0001) (Figure 1B) and total duration of attacks (F₆,₄₂=100.7, *p* < 0.0001) (Figure 1D) compared to vehicle (non-aggressive) and untreated *p*CPA groups. Moreover, there was marked increase in latency to attack in all treatment groups (F₆,₄₂=102.2, *p* < 0.0001) (Figure 1E) in comparison with untreated *p*CPA group. As previously observed, MOE treatment induced a behavioral response from day 1 while behavioral response in fluoxetine- and tryptophan-treated mice was observed on day 8 and 15 respectively. One-way ANOVA of the area under the curve (Figure 1F) showed that MOE (30, 100, 300 mg/kg) and fluoxetine significantly increased latency to attack compared to both vehicle and untreated *p*CPA groups (F₆,₄₂=102.4, *p* < 0.0001) while tryptophan showed significant increase compared to untreated *p*CPA group only.

We also evaluated the effect of the treatments on threat behaviors such as tail rattling and squeaking. We observed that MOE (30, 100, 300 mg/kg) significantly reduced the number of tail rattling (F₆,₄₂=10.94, *p* < 0.0001) (Figure 1G). The area under the curve for tail rattling showed significant reduction by MOE (30, 300 mg/kg) compared to vehicle and untreated *p*CPA groups (F₆,₄₂=10.94, *p* < 0.0001) (Figure 1H) while fluoxetine but not MOE (100 mg/kg) and tryptophan showed significant reduction in comparison with untreated *p*CPA mice. Moreover, all three treatment groups (MOE, fluoxetine, tryptophan) showed significant reduction in the number of squeaking (F₆,₄₂=29.05, *p* < 0.0001) (Figure 1K) from day 3–28. The *p*CPA-treated mice demonstrated increased squeaking behavior. The area under the curve for the graph of squeaking (F₆,₄₂=29.05, *p* < 0.0001) (Figure 1L) showed marked reduction following treatment with MOE (30, 100, 300 mg/kg), fluoxetine and tryptophan relative to vehicle and untreated *p*CPA groups. Also, MOE (30, 100, 300 mg/kg) significantly reduced pursuit duration (F₆,₄₂=15.29, *p* < 0.0001) (Figure 1I) from day 8–28 compared to untreated *p*CPA group. Considering the area under the curve, MOE (30, 100, 300 mg/kg) showed significant reduction in pursuit duration compared to both vehicle and untreated *p*CPA mice (F₆,₄₂=15.28, *p* < 0.0001) (Figure 1J) while fluoxetine and tryptophan showed significant reduction compared to untreated *p*CPA group only (*p* < 0.05).

### Antidepressant effect of MOE in mice

**FIGURE 2:**
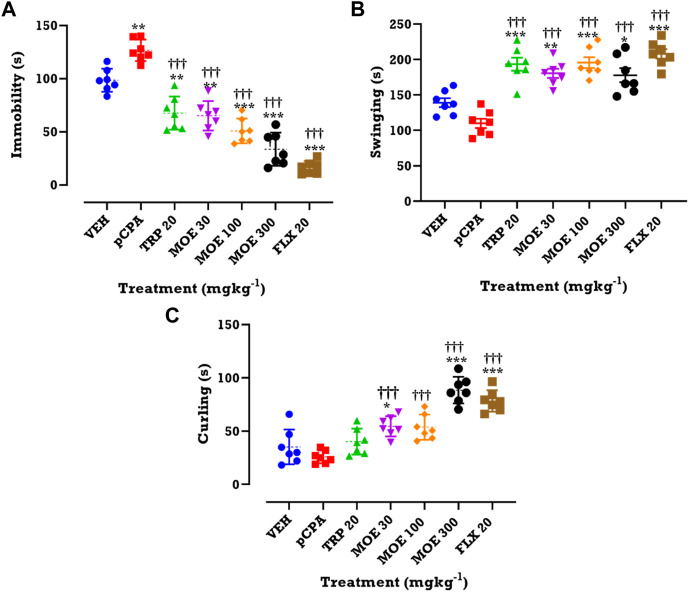
Effects of MOE, fluoxetine and tryptophan on **(A)** duration of immobility **(B)** duration of swinging and **(C)** duration of curling in the TST. Data are presented as group Means ± SEM (*n*=7). Significantly different from vehicle-treated group: ***p < 0.001, **p < 0.01, *p < 0.05; (One-way ANOVA followed by Tukey’s post hoc test). †††*p* < 0.001, significantly different from untreated *p*CPA group (One-way ANOVA followed by Tukey’s test).

Mice treated with *p*CPA also showed depressive behavior. This is supported by increase in immobility observed in the TST. This *p*CPA-induced depressive-like behavior was attenuated by MOE and fluoxetine after 28 days of treatment. Oral administration of MOE (30, 100, 300 mg/kg), tryptophan and fluoxetine significantly reduced duration of immobility compared to the vehicle and untreated *p*CPA groups (F₆,₄₂=44, *p* < 0.0001) (Figure 2A). Also, the same doses of MOE, tryptophan and fluoxetine significantly increased the duration of swinging in comparison with both vehicle and untreated *p*CPA mice (F₆,₄₂=20.95, *p* < 0.0001) (Figure 2B). In addition, MOE (30, 300 mg/kg) and fluoxetine but not tryptophan markedly increased duration of curling compared to vehicle and untreated *p*CPA groups (F₆,₄₂=27.14, *p* < 0.0001) (Figure 2C).

**FIGURE 3:**
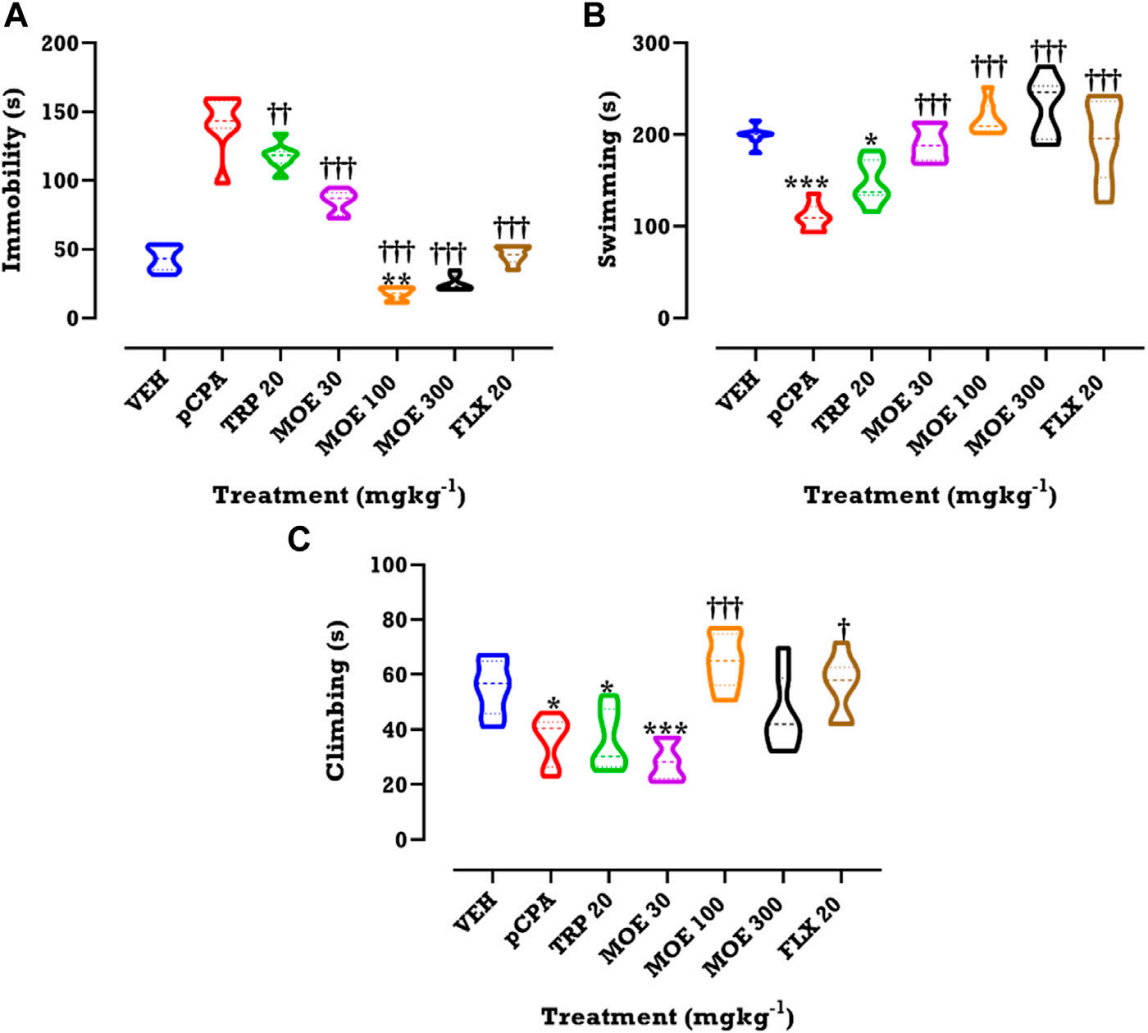
Effects of MOE, fluoxetine and tryptophan on **(A)** duration of immobility **(B)** duration of swimming and **(C)** climbing score in the FST. Data are presented as group Means ± SEM (*n*=7). Significantly different from vehicle: **p < 0.01, *p < 0.05; (One-way ANOVA followed by Tukey’s post hoc test). †††*p* < 0.001, ††*p* < 0.01, †*p* < 0.05; significantly different from untreated *p*CPA group (One-way ANOVA followed by Tukey’s post hoc test).

A *p*CPA-induced depression-like behavior was observed in the mice in the FST, being evidenced by increased immobility. This depressive-like behavior was abolished by MOE, tryptophan and fluoxetine treatment. MOE significantly reduced duration of immobility in comparison with both vehicle and untreated *p*CPA mice while fluoxetine and tryptophan markedly reduced duration of immobility relative to the untreated *p*CPA group only (F₆,₄₂=14, *p* < 0.0001) (Figure 3A). Interestingly, all the treatments did not significantly alter swimming and climbing score in comparison with vehicle group but MOE and fluoxetine significantly increased swimming duration compared to untreated *p*CPA mice (F₆,₄₂=18.82 and F6,42=11.54, *p* < 0.0001) (Figures 3B,C) respectively.

**FIGURE 4:**
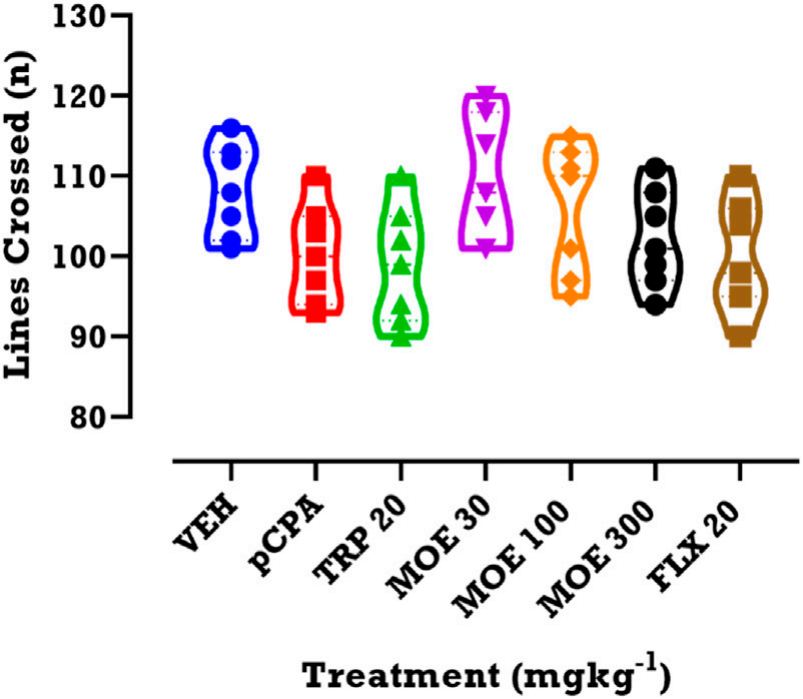
Effects of MOE, fluoxetine and tryptophan on lines crossed in the OFT. Data are presented as group Means ± SEM (n=7). Significantly different from vehicle-treated group: *p < 0.05, (One-way ANOVA followed by Tukey’s post hoc test).

We evaluated the impact of drug or extract treatment on locomotor activity. Compared to vehicle and untreated *p*CPA groups, we found that each of the three treatments had no significant effect on line crossing, which was used for estimating the locomotor activity (Figure 4; *p* > 0.05**).**

### Effect of MOE on weight variation

**FIGURE 5:**
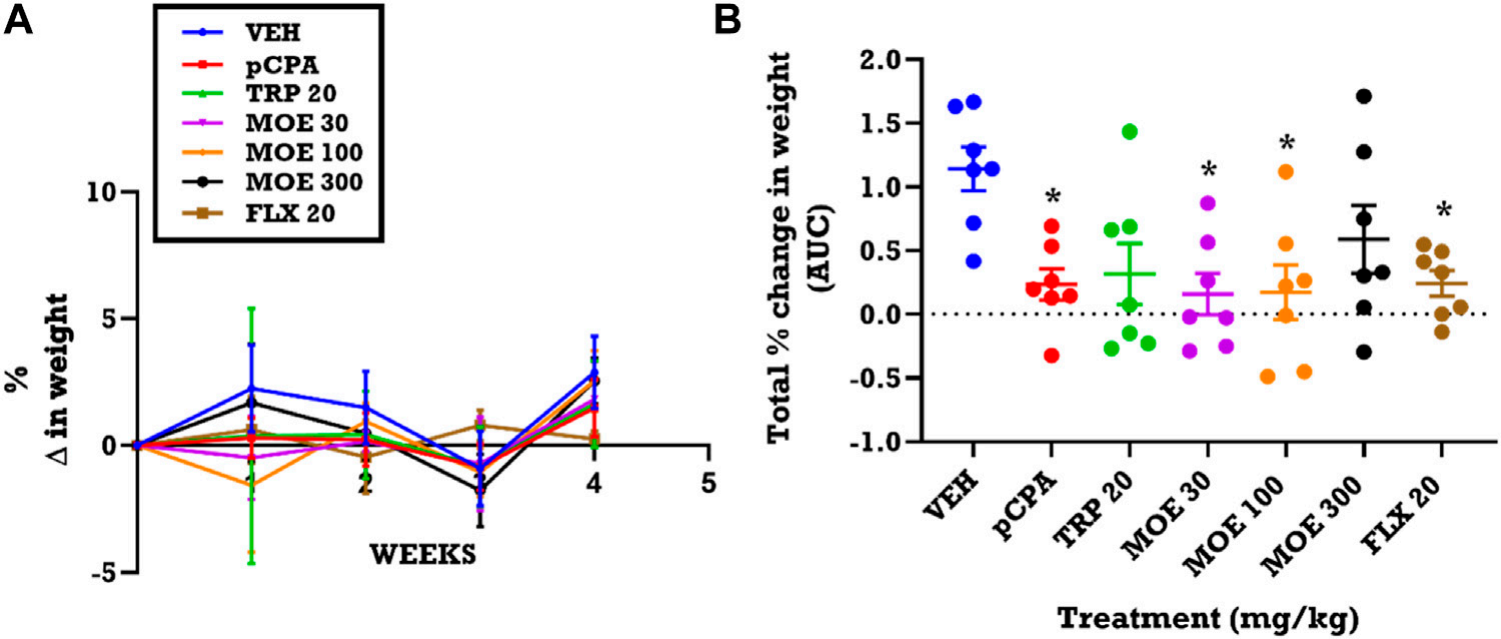
Effect of extract MOE (30, 100, 300 mg/kg), tryptophan 20 mg/kg and fluoxetine 20 mg/kg treatment on weight changes. Data are presented as both **(A)** a time course curve and **(B)** the Mean ± SEM of their AUCs (n=7). Significantly different from vehicle-treated group: *p < 0.05; (One-way ANOVA followed by Tukey’s post hoc test).

Since depression is normally associated with weight changes, we assessed the effect of treatment on weight variation. From the time-course graphs, we observed a slight increase in the weight of vehicle non-aggressive mice in the first week, followed by a reduction over the next 2 weeks and then a rise in weight in the last week of experiment (Figure 5A). This time-dependent variation seen in the vehicle group was not statistically different from mice that received *p*CPA, MOE, tryptophan, and fluoxetine. In contrast, analysis of the total weight variation for the 4 week period (presented as area under curves, AUCs) showed that MOE (300 mg/kg) and tryptophan reversed the *p*CPA-induced reduction in weight of the mice when compared with the vehicle group (Figure 5B). Conversely, lower doses of MOE (30, 100 mg/kg), and fluoxetine did not reverse the weight reduction induced by *p*CPA after the 28 days of treatment (Figure 5B).

### Effect of MOE on serotonin concentration in the brain

**FIGURE 6:**
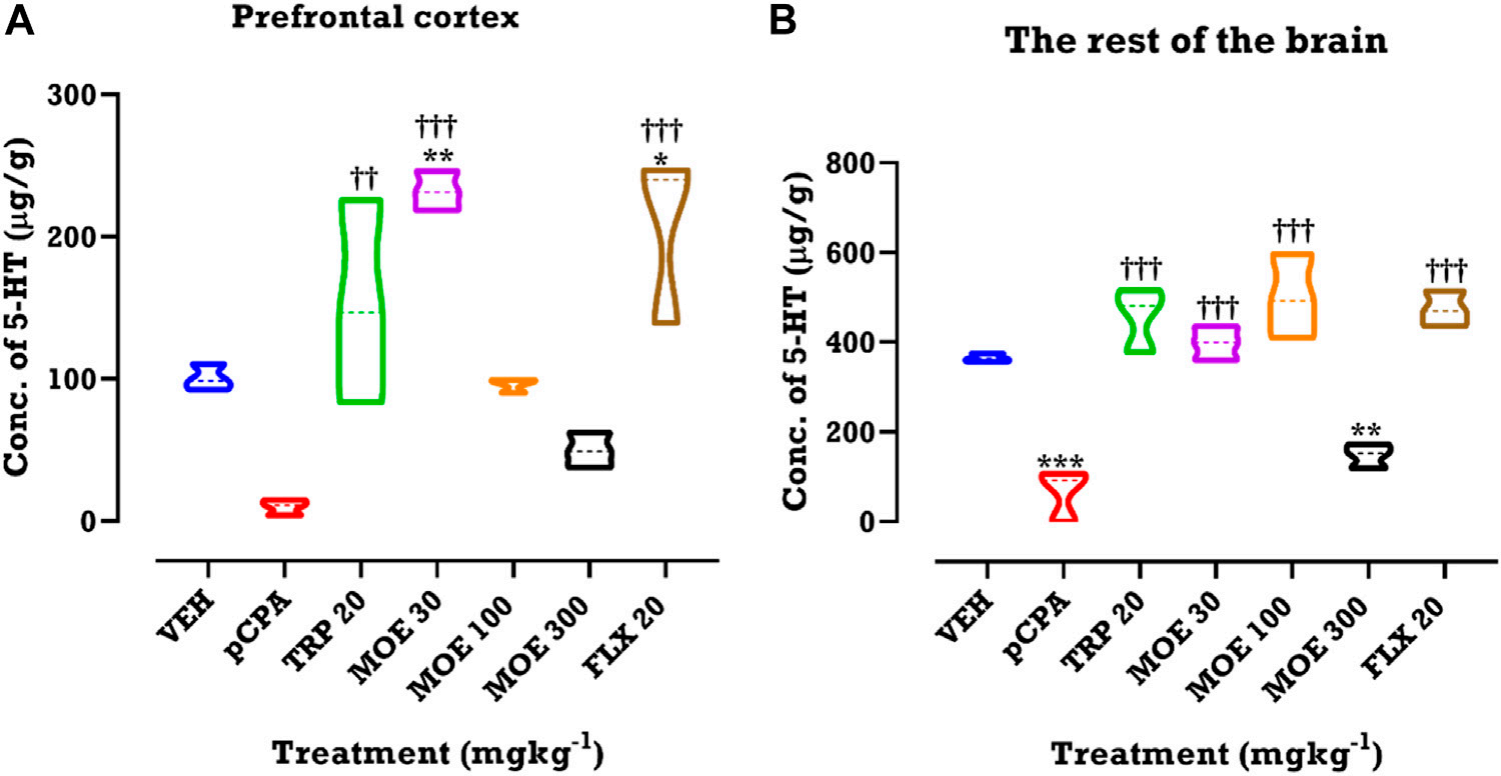
HPLC analysis of serotonin (5-hydroxytryptamine, 5-HT) in **(A)** prefrontal cortex and **(B)** rest of the brain regions of mice treated with MOE, fluoxetine and tryptophan. Data are presented as means ± SEM of 7 animals. Significant different from the vehicle-treated group; **p < 0.01, *p < 0.05: (One-way ANOVA followed by Tukey’s post hoc test). †††*p* < 0.001, ††*p* < 0.01; significantly different from untreated *p*CPA group (One-way ANOVA followed by Tukey’s test).

Since serotonin depletion in the brain has been linked with depression and aggression, we investigated the effect of MOE on the concentration of brain serotonin. We performed HPLC to measure serotonin concentrations after *p*CPA-induced serotonin depletion and subsequent treatment with MOE. We observed that *p*CPA slightly reduced serotonin concentration in the prefrontal cortex (PFC) and the rest of the brain. MOE, fluoxetine and tryptophan significantly reversed the *p*CPA-induced serotonin depletion in the PFC (F₆,₁₄ = 14.41, *p* < 0.0001; Figure 6A) and the rest of the brain regions (F₆,₁₄ = 28.51, *p* < 0.0001; Figure 6B). MOE (30 mg/kg)- and fluoxetine-induced increase in serotonin levels in the PFC was higher than vehicle group (*p* < 0.0001; Figure 6A). Higher doses of MOE (100, 300 mg/kg) slightly increased serotonin concentration in the PFC but did not reach statistical significance compared to the level in vehicle-treated mice (*p* > 0.05; Figure 6A). In the rest of the brain, low and medium doses of MOE produced higher serotonin concentration but not the high dose (Figure 6B) compared to untreated *p*CPA mice (*p* < 0 .0001; Figure 6B).

### Effect of MOE on dendritic spine density in the PFC

Depression and aggression induce significant changes in dendritic spine density, thus, we were interested in knowing if the anti-aggressive and antidepressant effect of MOE will reverse these changes. Z-stack images from our neurohistological examination of Golgi-spine in the PFC revealed the effect of MOE and the other treatments (fluoxetine and tryptophan) on dendritic branches and spine morphology.

**FIGURE 7:**
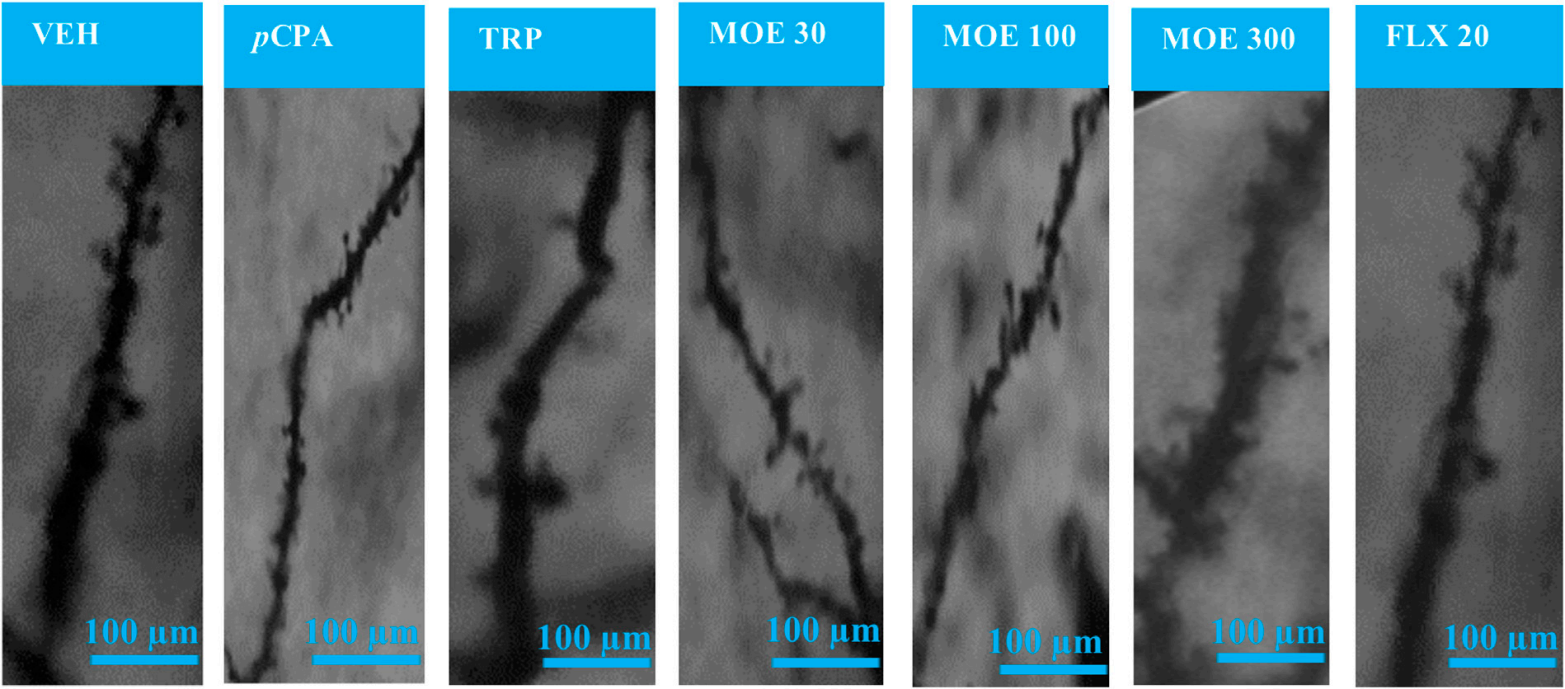
Representative images of Golgi-Cox-stained dendritic spines in the prefrontal cortex for each experimental group. VEH = Vehicle-treated group; *p*CPA = *p*CPA + vehicle group; TRP = Tryptophan-treated group; MOE 30, 100, 300 = *M. oppositifolius* extract-treated groups; FLX 20 = Fluoxetine-treated group.

**FIGURE 8:**
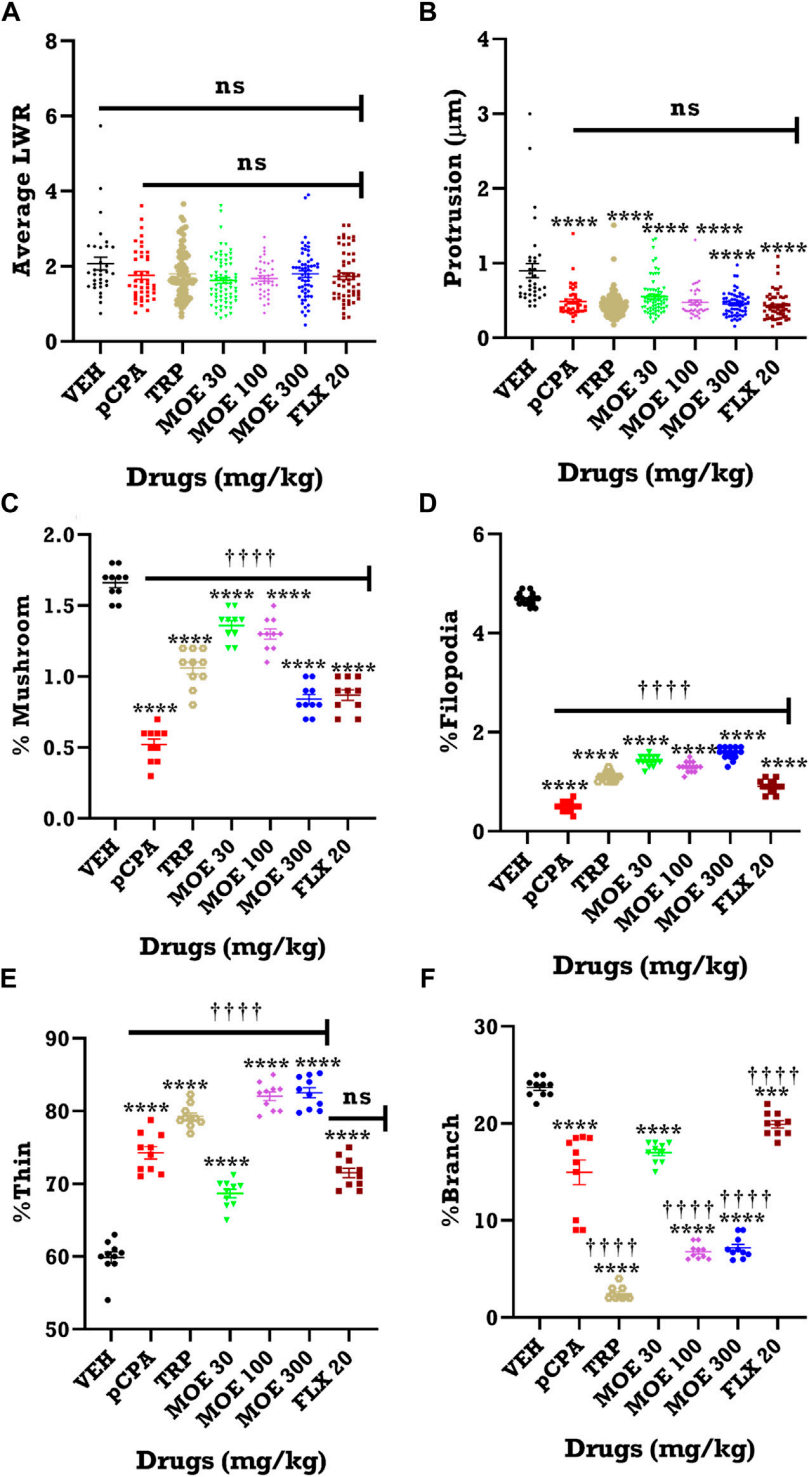
Quantification of dendritic spine from 10 labelled neurons from each group. Effect of MOE, fluoxetine and tryptophan on dendritic spine density in the prefrontal cortex of mice. The results are expressed as absolute values and expressed as mean ± SEM (*n* = 10). **p < 0.01 compared to vehicle-treated group; ††††*p* < 0.0001, ††*p* < 0.01; significantly different from *p*CPA + vehicle group (One-way ANOVA followed by Tukey’s test). **(A)** Average length to width ratio (LWR); **(B)** Protrusion; **(C)** % Mushroom spine density; **(D)** % Filopodia spine density; **(E)** % Thin spine density; **(F)** % Branched spine density.

The representative images of the different dendritic segments in the PFC in Figure 7 showed a reduced dendritic spine branches and density in the brain of untreated *p*CPA mice compared to vehicle group while MOE treatment produced increased dendritic spines (*p* < 0.05) (Figure 7; Table 1). Also, there was no significant difference in the average length-to-width ratio from the PFC for all the treatment groups (*p* > 0.05, Figure 8B) while protrusion density decreased significantly in MOE-treated mice compared to vehicle group (*p* < 0.0001, Figure 8B). We also observed that *p*CPA induced significant loss of matured mushroom-like spines and immature filopodia-like spines in the PFC (Figures 8C,D), which was reversed by MOE, tryptophan and fluoxetine (*p* < 0.0001, Figures 8C,D), although with a markedly lower density compared to vehicle group (*p* < 0.0001, Figures 8C,D). Moreover, analysis of the percentage thin-like spines also showed a significant increase in MOE-treated group relative to both vehicle and *p*CPA groups (*p* < 0.0001, Figure 8E) while fluoxetine showed no significant difference (*p* > 0.05, Figure 8E). Furthermore, MOE treatment resulted in a significant decrease in the percentage of branch-like spines compared to both vehicle and untreated *p*CPA mice (*p* < 0.0001, Figure 8F) while fluoxetine surprisingly showed a significant increase in comparison with untreated *p*CPA group (*p* < 0.001, Figure 8F).-

**TABLE 1:** Characteristics of dendritic spines in the prefrontal cortex regions of the mice of VEH: Vehicle; pCPA: pCPA + vehicle; TRP: Tryptophan 20 mg/kg; *Mallotus* oppositifolius extract (MOE 30, 100, 300 mg/kg) and FLX 20: Fluoxetine 20 mg/kg treatment group.

Spine characteristics	Treatment mg/kg (n=sample size)						
	VEH (n=430)	*p*CPA (n=330)	TRP 20 (n=950)	MOE 30 (n=690)	MOE 100 (n=400)	MOE 300 (n=620)	FLX 20 (n=570)
Mushroom (width >0.6 µm)	5(1.2%)	5(1.5%)	10(1.1%)	10(1.4%)	5(1.3%)	5(0.8%)	5(0.9%)
Filopodia (length >2 µm)	20(4.7%)	10(3%)	10(1.1%)	10(1.4%)	5(1.3%)	10(1.6%)	5(0.9%)
Stubby (LWR <1 µm)	10(2.3%)	30(9.1%)	40(4.2%)	60(8.7%)	30(7.5%)	50(8.1%)	30(5.3%)
Long thin (length <1 µm)	30(6.9%)	20(6.1%)	120(12.6%)	10(1.4%)	5(1.3%)	5(0.8%)	5(0.9%)
Thin (length <2 µm)	260(60.5%)	235(71.2%)	750(79%)	480(69.6%)	330(82.5%)	510(82.3%)	410(71.9%)

### Phytochemical fingerprint and constituents of MOE

**FIGURE 9:**
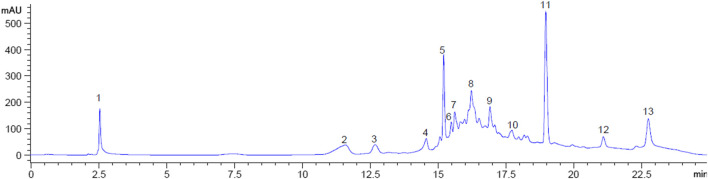
HPLC Fingerprint showing peaks at retention times for MOE. Numbers indicate individual peaks.

**FIGURE 10:**
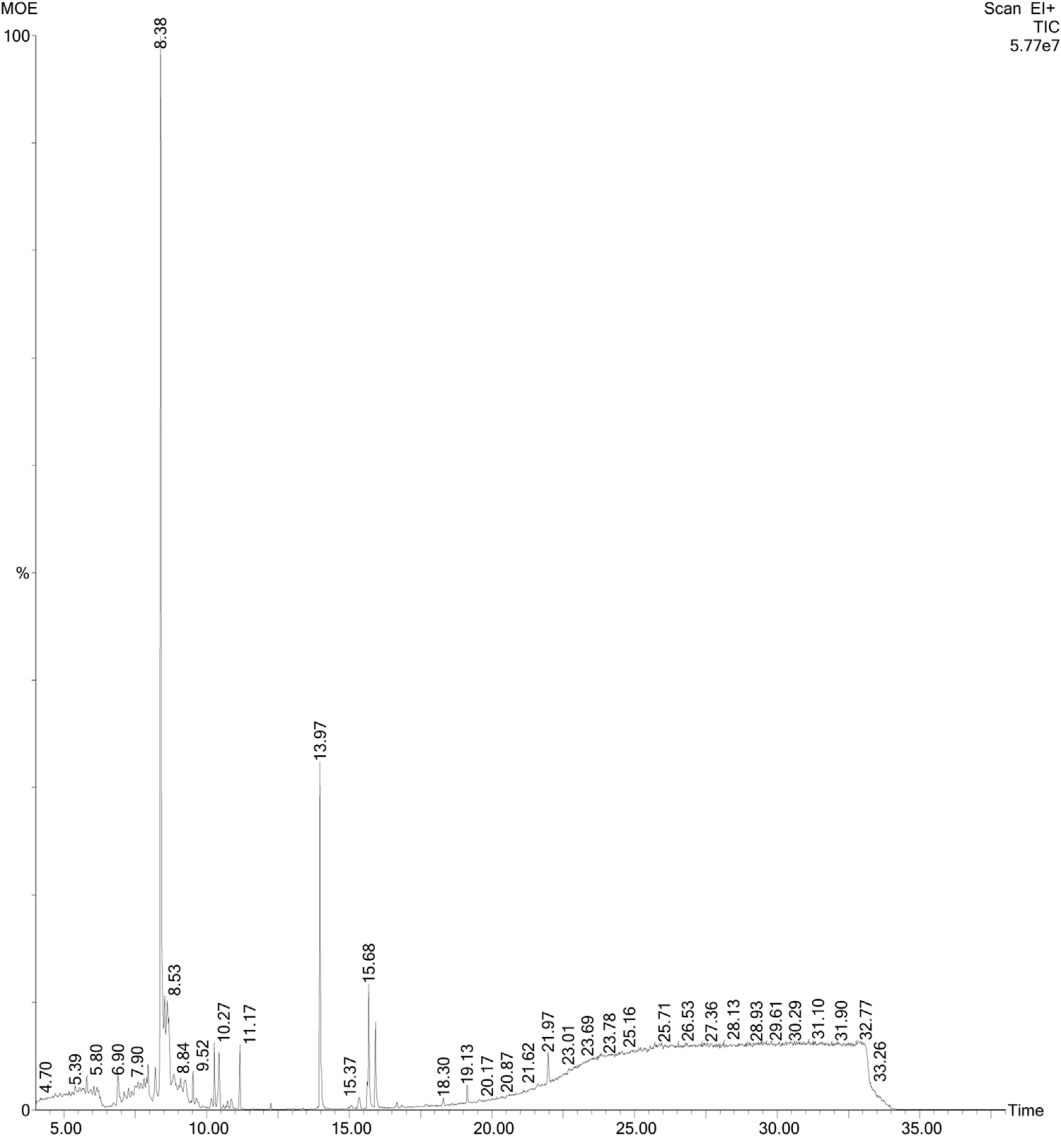
GC-MS chromatogram of MOE. Figure shows peaks of individual constituents at varying retention times, ranging from 4.70 to 33.24 min.

**FIGURE 11:**
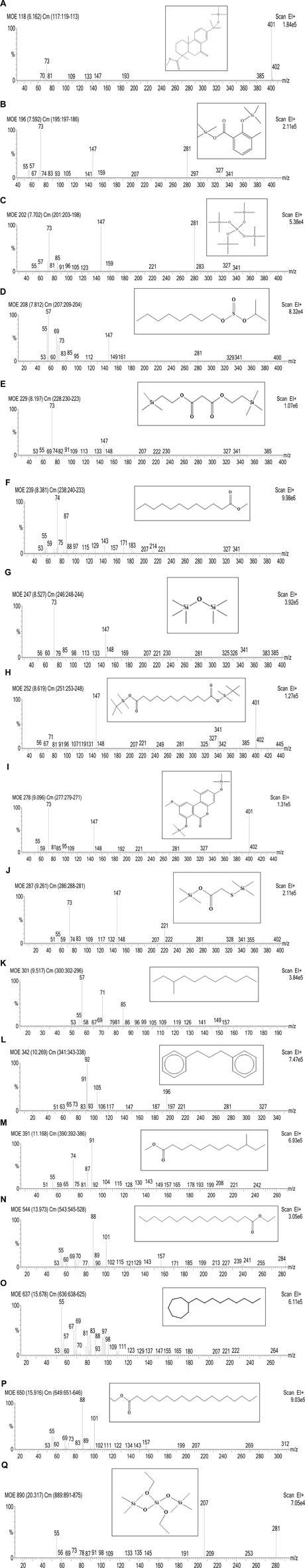
Mass spectra of seventeen phytocompounds in MOE identified by GC-MS analysis. **(A)** 15-Hydroxy-7-oxodehydroabietic acid, methyl ester, 15-trimethylsilyl ether with Retention Time (RT) 6.162 min; **(B)** Benzoic acid, 3-methyl-2-trimethylsilyloxy-, trimethylsilyl ester with Retention Time (RT) 7.592 min; **(C)** Trisiloxane, 1,1,1,5,5,5-hexamethyl-3,3-bis [(trimethylsilyl) oxy] with Retention Time (RT) 7.702 min; **(D)** Sulfurous acid, octyl 2-propyl ester with Retention Time (RT) 7.812 min; **(E)** Malonic acid, bis (2-trimethylsilylethyl ester) with Retention Time (RT) 8.197 min; **(F)** Dodecanoic acid, methyl ester with Retention Time (RT) 8.381 min; **(G)** Hexamethyl- Disiloxane with Retention Time (RT) 8.527 min; **(H)** Dodecanedioic acid, bis(tert-butyldimethylsilyl) ester with Retention Time (RT) 8.619 min; **(I)** 3,7-Bis [(trimethylsilyl) oxy]-9-methoxy-1-methyl (6H) dibenzo [b,d] pyran-6-one with Retention Time (RT) 9.096 min; **(J)** Mercaptoacetic acid, bis(trimethylsilyl) with Retention Time (RT) 9.261 min; **(K)** Undecane, 3-methyl- with Retention Time (RT) 9.517 min; **(L)** Benzene, 1,1′-(1, 3-propanediyl) bis- with Retention Time (RT) 10.269 min; **(M)** Methyl 10-phenyldecanoate with Retention Time (RT) 11.168 min; **(N)** Hexadecenoic acid, ethyl ester with Retention Time (RT) 13.973 min; **(O)** 1-Nonylcycloheptane with Retention Time (RT) 15.678 min; **(P)** Octadecanoic acid, ethyl ester with Retention Time (RT) 15.916 min; **(Q)** Silicic acid, diethyl bis(trimethylsilyl) ester with Retention Time (RT) 20.317 min.

From an initial weight of 600 g powdered plant material, 13.08 g (representing 2.18% yield) of MOE was obtained. Preliminary qualitative phytochemical screening of MOE revealed the presence of saponins, phenolic compounds, reducing sugars, alkaloids and phytosterols. General profiling of crude MOE eluted 13 separate HPLC peaks based on their adsorption and partition. The retention times were between 2.5 and 23 min **(** Figure 9). GC-MS chromatogram showed 17 phytochemical constituents of MOE (Figure 10; Table 2). The major identified compounds include 15-Hydroxy-7-oxodehydroabietic acid, methyl ester, 15-trimethylsilyl ether (Figure 11A); Benzoic acid, 3-methyl-2-trimethylsilyloxy-, trimethylsilyl ester (Figure 11B); Trisiloxane, 1,1,1,5,5,5-hexamethyl-3,3-bis [(trimethylsilyl)oxy](tetratrimethylsilyl silicate) (Figure 11C); Sulfurous acid, octyl 2-propyl ester (Figure 11D); Malonic acid, bis (2-trimethylsilylethyl ester) (Figure 11E); dodecanoic acid methyl ester (methyl laurate) (Figure 11F); Hexamethyl- Disiloxane (Figure 11G); Dodecanedioic acid, bis(tert-butyldimethylsilyl) ester (Figure 11H); 3,7-Bis [(trimethylsilyl)oxy]-9-methoxy-1-methyl (6H) dibenzo [b,d]pyran-6-one (Figure 11I); Mercaptoacetic acid, bis(trimethylsilyl) (Figure 11J); undecane 3-methyl (hendecane) (Figure 11K benzene 1,1’-(1, 3-propanediyl) bis (Figure 11L); methyl 10-phenyldecanoate (decanoic acid) (Figure 11M); hexadecenoic acid ethyl ester (ethyl palmitate) (Figure 11N); 1-nonylcycloheptane (cycloheptane) (Figure 11O); Octadecanoic acid, ethyl ester (ethyl stearate) (Figure 11P); Silicic acid, diethyl bis(trimethylsilyl) ester (Figure 11Q). These constituent compounds and their potential role in the anti-aggressive effect of MOE are discussed in the next section.

**TABLE 2:** List of compounds present in the methanol leaf extract of *Mallotus oppositifolius* identified by GC-MS analysis.

No.	Retention time (min)	Phytochemical compound	Molecular formula	Molecular weight (g/mol)	% Area
1	6.162	15-Hydroxy-7-oxodehydroabietic acid, methyl ester,15-trimethylsilyl ether	C_24_H_36_O_4_Si	416.60	2.11
2	7.592	Benzoic acid, 3-methyl-2-trimethylsilyloxy-, trimethylsilyl ester	C_14_H_24_O_3_Si_2_	296.51	2.15
3	7.702	Trisiloxane, 1,1,1,5,5,5-hexamethyl-3,3-bis [(trimethylsilyl)oxy]-	C_12_H_36_O_4_Si_5_	384.84	1.20
4	7.812	Sulfurous acid, octyl 2-propyl ester	C_11_H_24_O_3_S	236.37	1.13
5	8.197	Malonic acid, bis (2-trimethylsilylethyl ester)	C_13_H_28_O_4_Si_2_	304.53	1.40
6	8.381	Dodecanoic acid, methyl ester	C_13_H_26_O_2_	214.34	34.92
7	8.527	Hexamethyl- Disiloxane	C_6_H_18_OSi_2_	162.38	4.20
8	8.619	Dodecanedioic acid, bis(tert-butyldimethylsilyl) ester	C_24_H_50_O_4_Si_2_	458.80	8.69
9	9.096	3,7-Bis [(trimethylsilyl)oxy]-9-methoxy-1-methyl (6H) dibenzo [b,d]pyran-6-one	C_21_H_28_O_5_Si_2_	416.60	1.96
10	9.261	Mercaptoacetic acid, bis(trimethylsilyl)	C_8_H_20_O_2_SSi_2_	236.48	2.00
11	9.517	Undecane, 3-methyl-	C_12_H_26_	170.33	0.84
12	10.269	Benzene, 1,1’-(1, 3-propanediyl) bis-	C_15_H_16_	196.29	1.49
13	11.168	Methyl 10-phenyldecanoate	C_14_H_28_O_2_	228.37	1.44
14	13.973	Hexadecenoic acid, ethyl ester (Ethyl palmitate)	C_18_H_36_O_2_	284.50	9.95
15	15.678	1-Nonylcycloheptane	C_16_H_32_	224.42	4.22
16	15.916	Octadecanoic acid, ethyl ester	C_20_H_40_O_2_	312.50	2.55
17	20.317	Silicic acid, diethyl bis(trimethylsilyl) ester	C_10_H_28_O_4_Si_3_	296.58	1.05

## Discussion

The present study establishes a murine model of depression-related aggression in which *Mallotus oppositifolius* extract (MOE) exerted anti-aggressive effects *via* increased serotonin (5-HT) concentration and dendritic spine density in the prefrontal cortex (PFC). In using this model, we showed that MOE produced a more rapid decline in aggressive behavior in *p*CPA-aggravated aggression in mice compared to mice treated with fluoxetine (conventional antidepressant) and tryptophan. In addition, MOE did not produce any significant change in locomotor activity, which could have confounded the antidepressant effect observed. Finally, we demonstrated that the highest dose of MOE reversed weight reduction induced by *p*CPA.

### MOE reduced aggressive and depressive behavior in mice

Depression has been strongly linked to aggression and suicide (WHO, 2018; Locci and Pinna, 2019). In the present study, we showed that *p*CPA induces depression and aggression in mice. The depression-like behavior was evidenced by increased duration of immobility in the tail suspension test (TST) and forced swimming test (FST) while aggression was characterized by decreased latency to first attack, increased total number of attacks, attack duration, tail rattling, pursuit duration and number of squeaking in the resident mice. Our observations are consistent with the report by Studer et al. (2015) who demonstrated that *p*CPA (300 mg/kg for 3 consecutive days) increased the total duration of attack, decreased attack latency and increased duration of social behavior spent on attack. Indeed, 5-HT deficiency has been reported as an antecedent to depression and aggression (Manchia et al., 2017). Hence, it was not surprising that *p*CPA, which depletes serotonin in the brain by irreversibly inhibiting 5-HT synthesis, produced aggression and depression in the present study. Interestingly, MOE significantly decreased duration of immobility in the TST and FST, indicating antidepressant-like effects. This is in line with our previous murine model in which we reported antidepressant potential of MOE (Kukuia et al., 2014). In addition, MOE treatment in the present study also reversed *p*CPA-induced aggression by increasing latency to attack, and significantly reducing number and duration of attacks, number of tail rattling, pursuit duration and number of squeaking in aggressive mice. Unlike fluoxetine and tryptophan whose anti-aggressive effects were observed on day 8 following treatment, the anti-aggressive effect of MOE began on the first day of treatment, suggesting a more rapid effect. This result is consistent with our previous finding that showed that MOE exerted a more rapid-antidepressant effect compared to conventional antidepressants such as imipramine and fluoxetine (Kukuia et al., 2016b). Also, the present study showed that MOE and fluoxetine significantly increased duration of swinging and swimming in the TST and FST respectively, suggesting their interaction with serotoninergic pathway. It is worth noting that MOE produced these effects without compromising locomotor activity as observed in the open-field test, a reliable test for studying locomotion and anxiety-related behavior (Kraeuter et al., 2019b).

Therefore, we can infer that the antidepressant effects of MOE were not as a result of any psychomotor stimulant effect. Moreover, our study proves that MOE possesses both antidepressant and fast-acting anti-aggressive effects in mice.

### The anti-aggressive effect of MOE is *via* serotoninergic pathway

Neurotransmitters such as 5-HT in various brain regions, particularly the PFC, are implicated in depressive, aggressive and suicidal behaviors (Desmyter et al., 2011; van Heeringen et al., 2011). Neurobiologically, increased 5-HT activity in the brain decreases aggression in murine resident-intruder test and attenuates aggressive and suicidal behaviors in humans (Yanowitch and Coccaro, 2011; Wang et al., 2019). Furthermore, 5-HT contributes to the neuronal mechanisms in the treatment of aggressive and suicidal behaviors (Quadros et al., 2020). In the present study, *p*CPA depleted brain 5-HT concentration and induced aggression in the animals, which aligns with previous studies (Coccaro et al., 2015; Mann, 2013). Our study revealed that MOE significantly reversed the *p*CPA-induced 5-HT depletion in the PFC and other brain regions. These findings suggest that the anti-aggressive effect of MOE was at least in part, mediated via the serotoninergic pathway, and support recent studies in which higher 5-HT concentration in the brain showed anti-aggressive effects in animals and humans (Boldrini and Mann, 2015; Quadros et al., 2020). It is important to note that tryptophan, a precursor for 5-HT synthesis, showed anti-aggressive effect and increased 5-HT levels in the brain (Kulikov et al., 2012). Also, fluoxetine, a selective serotonin reuptake inhibitor, increased 5-HT concentration in the PFC and other brain regions in the present study, which confirms the role of the serotoninergic pathway in reducing aggressive behaviors. These findings corroborate previous studies that showed fluoxetine reverses offensive aggression by increasing 5-HT levels in the brain (Coccaro et al., 2015). In summary, serotoninergic pathway partly mediates the anti-aggressive effect of MOE.

### MOE reversed *p*CPA-Induced loss of dendritic spine density

Although, neuronal mechanisms and brain regions that mediate aggression and suicide are not completely understood, the PFC has been described to be pivotal in aggressive and suicidal tendencies (Boldrini and Mann, 2015). The present study revealed that MOE significantly reversed *p*CPA-induced dendritic spine loss in the mice PFC after 4 weeks of treatment. Dendritic spines are tiny protrusions from the surface of neurons, which are considered sites of cortical excitatory synapses (Kasai et al., 2003). Alterations in dendritic spines are considered the basis of neuroplasticity (i.e. the ability of the neural network to change and adapt) and have been shown to regulate mood and behavior *via* serotoninergic pathway (de Bartolomeis et al., 2014; Alenina and Klempin, 2015). Moreover, neurogenesis and synaptic plasticity are critical in the management of psychiatric disorders, aggression and suicidal behaviors. A previous study indicated that the inhibitory neurons and interneurons found in the PFC modulate mood, thoughts and actions (Ohira et al., 2013), and that abnormalities or reduction in these neuronal cells always precede severe psychiatric disorders and suicide (Luscher et al., 2011). The effect of MOE on dendritic spine changes in the present study may therefore suggest that neurogenesis and/or synaptic plasticity contribute to the anti-aggressive effect of MOE. We can deduce that *p*CPA-associated reduction in 5-HT concentration may explain the alteration in dendritic spine density. It is worth noting that *p*CPA significantly reduced the mushroom and filopodia dendritic spines in the mouse PFC in our study. The mushroom spines represent long-lived dendritic spines usually observed in mature neurons, which regulate behavioral patterns (Berry and Nedivi, 2017; Helm et al., 2021), and their diminution may explain the aggressive behavior exhibited (Anilkumar et al., 2021). It might also imply that a decrease in 5-HT caused mature neurons to die, which resulted in the reduction of mushroom spines (Popova and Naumenko, 2019). On the other hand, the filopodia spines, usually considered the precursors for the other dendritic spines, are substrates for activity-related growth and strengthening of spines (Kasai et al., 2003; Ozcan, 2017). Also, the findings that *p*CPA reduced the mushroom and filopodia spines may suggest decline in neurogenesis and/or synaptic plasticity (Chang et al., 2018; Popova and Naumenko, 2019). Interestingly, MOE and fluoxetine reversed the loss of mushroom and filopodia spines induced by *p*CPA in the present study. This is consistent with other studies which demonstrated that chronic fluoxetine treatment increased cell proliferation in the PFC of adult rodents (Czéh et al., 2007; Hodes et al., 2010). In addition, we observed that MOE and tryptophan, but not fluoxetine, increased the number of tiny, thin spines in mice treated with pCPA, which strongly suggests neurogenesis. The thin spines are considered immature spines and are predominant during development. It is possible that the MOE and tryptophan increased the development of new dendrites, hence the increase in immature spines. Although not proven in this study, we are of the view that the seemingly lack of significant difference in the thin spines in fluoxetine-treated mice may be due to the short duration of treatment (4 weeks). This assertion is supported by a previous preclinical study that found increased neuronal cells in the PFC after 6 weeks of fluoxetine treatment but not after 3 weeks (Ohira et al., 2013). Also, it has been shown that selective serotonin reuptake inhibitors such as fluoxetine, as used in the present study, exert their effects secondary to neurogenesis and synaptic plasticity, and that it takes about 6 weeks for neurons to mature (Kodama et al., 2004). On the whole, MOE prevented loss of dendritic spine density and contributed to neurogenesis and/or neuroplasticity, which partly underlie its anti-aggressive effect.

### Constituent compounds of MOE and their potential anti-aggressive effect

Since phyto-components are responsible for the biological actions, it is vital to characterize the MOE’s contents prior to drug discovery. Our HPLC examination of the MOE eluted thirteen distinct peaks that will serve as a reference and quality control for future research. We identified 17 compounds in the MOE using gas chromatography-mass spectrometry, including methyl laurate; ethyl palmitate; ethyl stearate; 15-hydroxy-7-oxodehydroabietic acid, methyl ester,15-trimethylsilyl ether; benzoic acid, 3-methyl-2-trimethylsilyloxy-, trimethylsilyl ester; tetratrimethylsilyl silicate; -9-methoxy-1-methyl (6H) dibenzo [b,d]pyran-6-one; bis(trimethylsilyl) mercaptoacetic acid; 3-methyl undecane; benzene 1,1'-(1,3-propanediyl) bis; methyl 10-phenyldecanoate; cyclohexane; silicic acid and diethyl bis(trimethylsilyl) ester. Intriguingly, some of the discovered chemicals exhibit biological activities that imply they contribute to the anti-aggressive effect of MOE found in our study. In the brain of mice, phenolic substances such as tetratrimethylsilyl silicate have antioxidant, neuroprotective, and antidepressant properties (Khan et al., 2019; Dalmagro et al., 2020; Sherif, 2021). In addition, the ester derivatives found in MOE may induce neurogenesis, and these components may be responsible for the enhanced dendritic spine density in the PFC seen in this study (Kawakita et al., 2006). In addition, we hypothesize that the presence of methyl laurate, the most abundant component of the extract, may considerably contribute to a reduction in aggressive behavior. This claim is supported by evidence that methyl laurate works as a pheromone signal in Drosophila and promotes mating-related attraction and social engagement (Dweck et al., 2015). We believe that the increased attraction induced by methyl laurate, a known pheromone, contributes to the decreased aggressiveness observed in mice treated with MOE. In fact, more pheromones have been demonstrated to improve mood. 3b-androsta-4,16-diene-3-ol, an unique investigational nasal medication that acts on pheromone pathways, was discovered to have a soothing impact on people with social anxiety disorder (Liebowitz et al., 2014). This further supports our theory that methyl laurate may have contributed to the observed behavioral effect of MOE. Furthermore, we hypothesize that the presence of methyl laurate in MOE may facilitate the diffusion of additional beneficial phyto-constituents into the brain. Based on a recent observation that a comparable chemical, ethyl laurate, increased intranasal diazepam delivery to the brain of rabbits, this assumption is true (Li et al., 2002). The most common fatty acid in the brain, ethyl palmitate, and benzoic acid compounds found in MOE have anti-inflammatory effects (Kumar et al., 2003; Saeed et al., 2012). Considering the association between inflammation and depression (Benedetti et al., 2020), we believe that these derivatives likely contributed to the observed behavioral effects. In addition, a recent study demonstrated that ethyl stearate, isolated from *Plastrum testudinis* and also present in MOE, increased the expression of tyrosine hydroxylase (an enzyme required for dopamine synthesis) and inhibited alpha-synuclein in rats, indicating a potential neuroprotective effect in Parkinson’s disease (Ye et al., 2021). Our contention is that this impact may also be advantageous in the treatment of depression, as dopamine also modulates mood (Dujardin and Sgambato, 2020). In addition, Igwe et al. (2016) found glutaconic anhydride, n-hexadecanoic acid, 3-methyl butanoic acid, valeric acid, sorbic acid, and oleamide in MOE, and some of these compounds were validated in our extract. Noting that the pharmacological effects of additional compounds in MOE have not been fully explained, we propose that future studies consider isolating the compounds found in our work and investigating their probable role in the depressive and anti-aggressive properties of MOE.

## Conclusion

To the best of our knowledge, we have provided the first experimental evidence showing that MOE exhibits antidepressant and anti-aggressive effects by increasing serotonin levels and dendritic spine density in the PFC. The findings from our study suggest that MOE may possess suicide-reducing potential or reduce depression-associated aggressive behaviors. The resent study is topical and timely, and may provide additional pharmacotherapeutic option in the prevention, treatment or management of depression-associated aggressive behaviors. The effect of the extract on the role of inflammatory mediators (specifically IL-2, IL-6, IL-8 and TNF-ɑ) in aggression and depression could be considered for future research.

## Data Availability

The raw data supporting the conclusion of this article will be made available by the authors, without undue reservation.

## References

[B1] Alagappan V. Shah R. Meisner R. Research H. (2020). The effects of nutrients on stress and aggression: Integrative approaches to behavioral and emotional modification. J. Gastroenterol. Hepatol. Res. 9 (6), 3362–3366. 10.17554/j.issn.2224-3992.2020.09.975

[B2] Alenina N. Klempin F. (2015). The role of serotonin in adult hippocampal neurogenesis. Behav. Brain Res. 277, 49–57. 10.1016/j.bbr.2014.07.038 25125239

[B3] American Veterinary Medical Association (2013). AVMA guidelines for the euthanasia of animals: 2013 edition. Schaumburg, IL: American Veterinary Medical Association.

[B4] Anilkumar S. Patel D. de Boer S. F. Chattarji S. Buwalda B. (2021). Decreased dendritic spine density in posterodorsal medial amygdala neurons of proactive coping rats. Behav. Brain Res. 397, 112940. 10.1016/j.bbr.2020.112940 33126115

[B5] Benedetti F. Aggio V. Pratesi M. L. Greco G. Furlan R. (2020). Neuroinflammation in bipolar depression. Front. Psychiatry 11, 71. 10.3389/fpsyt.2020.00071 32174850 PMC7054443

[B6] Berry K. P. Nedivi E. (2017). Spine dynamics: Are they all the same? Neuron 96 (1), 43–55. 10.1016/j.neuron.2017.08.008 28957675 PMC5661952

[B8] Boldrini M. Mann J. J. (2015). “Depression and suicide,” in Neurobiology of brain disorders: Biological basis of neurological and psychiatric disorders (Elsevier), 709–729. 10.1016/B978-0-12-398270-4.00043-4

[B86] Brent D. (2009). In search of endophenotypes for suicidal behavior. Am. J. Psychiatry 166 (10), 1087–1089. 10.1176/appi.ajp.2009.09081131 19797437

[B9] Burkill H. M. (1994). The useful plants of West Tropical Africa, Vol 2. Families E-I. Richmond, United Kingdom: Royal Botanic Gardens, Kew.

[B10] Çetin F. H. Torun Y. T. Güney E. (2017). “The role of serotonin in aggression and impulsiveness,” in Serotonin: A chemical messenger between all types of living cells, 241.

[B11] Chang W. H. Lee I. H. Chi M. H. Lin S.-H. Chen K. C. Chen P. S. (2018). Prefrontal cortex modulates the correlations between brain-derived neurotrophic factor level, serotonin, and the autonomic nervous system. Sci. Rep. 8 (1), 2558–2559. 10.1038/s41598-018-20923-y 29416077 PMC5803248

[B12] Christensen C. B. Soelberg J. Stensvold C. R. Jäger A. K. (2015). Activity of medicinal plants from Ghana against the parasitic gut protist Blastocystis. J. Ethnopharmacol. 174, 569–575. 10.1016/j.jep.2015.03.006 25773490

[B87] Coccaro E. F. Fanning J. R. Phan K. L. Lee R. (2015). Serotonin and impulsive aggression. CNS Spectrums 20 (3), 295–302. 10.1017/S1092852915000310 25997605

[B14] Czéh B. Müller-Keuker J. I. H. Rygula R. Abumaria N. Hiemke C. Domenici E. (2007). Chronic social stress inhibits cell proliferation in the adult medial prefrontal cortex: Hemispheric asymmetry and reversal by fluoxetine treatment. Neuropsychopharmacology 32 (7), 1490–1503. 10.1038/sj.npp.1301275 17164819

[B15] Dalmagro A. P. Camargo A. Pedron N. B. Garcia S. A. Zeni A. L. B. (2020). Morus nigra leaves extract revokes the depressive-like behavior, oxidative stress, and hippocampal damage induced by corticosterone: A pivotal role of the phenolic syringic acid. Behav. Pharmacol. 31 (4), 397–406. 10.1097/FBP.0000000000000549 32040015

[B16] Das G. Reuhl K. Zhou R. (2013). “The golgi–cox method,” in Neural development (Springer), 313–321. 10.1007/978-1-62703-444-9_29 23681640

[B17] de Bartolomeis A. Latte G. Tomasetti C. Iasevoli F. (2014). Glutamatergic postsynaptic density protein dysfunctions in synaptic plasticity and dendritic spines morphology: Relevance to schizophrenia and other behavioral disorders pathophysiology, and implications for novel therapeutic approaches. Mol. Neurobiol. 49 (1), 484–511. 10.1007/s12035-013-8534-3 23999870

[B18] Desmyter S. van Heeringen C. Audenaert K. (2011). Structural and functional neuroimaging studies of the suicidal brain. Prog. Neuropsychopharmacol. Biol. Psychiatry 35 (4), 796–808. 10.1016/j.pnpbp.2010.12.026 21216267

[B20] Dujardin K. Sgambato V. (2020). Neuropsychiatric disorders in Parkinson’s disease: What do we know about the role of dopaminergic and non-dopaminergic systems? Front. Neurosci. 14, 25. 10.3389/fnins.2020.00025 32063833 PMC7000525

[B21] Dweck H. K. Ebrahim S. A. Thoma M. Mohamed A. A. Keesey I. W. Trona F. (2015). Pheromones mediating copulation and attraction in Drosophila. Proc. Natl. Acad. Sci. U. S. A. 112 (21), E2829–E2835. 10.1073/pnas.1504527112 25964351 PMC4450379

[B23] Friard O. Gamba M. (2016). Boris: A free, versatile open-source event-logging software for video/audio coding and live observations. Methods Ecol. Evol. 7 (11), 1325–1330. 10.1111/2041-210X.12584

[B24] Fritz M. Shenar R. Cardenas-Morales L. Jäger M. Streb J. Dudeck M. (2020). Aggressive and disruptive behavior among psychiatric patients with major depressive disorder, schizophrenia, or alcohol dependency and the effect of depression and self-esteem on aggression. Front. Psychiatry 11, 599828. 10.3389/fpsyt.2020.599828 33343427 PMC7744284

[B45] Hammad T. A. Laughren T. Racoosin J. (2006). Suicidality in pediatric patients treated with antidepressant drugs. Arch. Gen. Psychiatry 63 (3), 332–339. 16520440 10.1001/archpsyc.63.3.332

[B89] Hawton K. Casañas I. Comabella C. Haw C. Saunders K. (2013). Risk factors for suicide in individuals with depression: A systematic review. J. Affect. Disord. 147 (1–3), 17–28. 10.1016/j.jad.2013.01.004 23411024

[B27] Helm M. S. Dankovich T. M. Mandad S. Rammner B. Jähne S. Salimi V. (2021). A large-scale nanoscopy and biochemistry analysis of postsynaptic dendritic spines. Nat. Neurosci. 24 (8), 1151–1162. 10.1038/s41593-021-00874-w 34168338

[B28] Hodes G. E. Hill-Smith T. E. Suckow R. F. Cooper T. B. Lucki I. (2010). Sex-specific effects of chronic fluoxetine treatment on neuroplasticity and pharmacokinetics in mice. J. Pharmacol. Exp. Ther. 332 (1), 266–273. 10.1124/jpet.109.158717 19828877 PMC2802485

[B29] Hosanagar A. Schmale A. LeBlanc A. (2021). Ketamine's rapid antisuicidal effects are not attenuated by Buprenorphine. J. Affect. Disord. 282, 252–254. 10.1016/j.jad.2020.12.120 33418374

[B30] Igwe K. Madubuike A. Otuokere I. Amaku F. (2016). GC-MS analysis for structural identification and bioactive compounds in methanolic leaf extract of Mallotus oppositifolius. Int. J. Sci. Res. Manag. 4 (5). 10.18535/ijsrm/v4i5.04

[B31] Kadriu B. Musazzi L. Henter I. D. Graves M. Popoli M. Zarate C. A. (2019). Glutamatergic neurotransmission: Pathway to developing novel rapid-acting antidepressant treatments. Int. J. Neuropsychopharmacol. 22 (2), 119–135. 10.1093/ijnp/pyy094 30445512 PMC6368372

[B32] Kasai H. Matsuzaki M. Noguchi J. Yasumatsu N. Nakahara H. (2003). Structure–stability–function relationships of dendritic spines. Trends Neurosci. 26 (7), 360–368. 10.1016/S0166-2236(03)00162-0 12850432

[B33] Kawakita E. Hashimoto M. Shido O. (2006). Docosahexaenoic acid promotes neurogenesis *in vitro* and *in vivo* . Neuroscience 139 (3), 991–997. 10.1016/j.neuroscience.2006.01.021 16527422

[B34] Khan K. Najmi A. K. Akhtar M. (2019). A natural phenolic compound quercetin showed the usefulness by targeting inflammatory, oxidative stress markers and augment 5-HT levels in one of the animal models of depression in mice. Drug Res. 69 (07), 392–400. 10.1055/a-0748-5518 30296804

[B35] Klonsky E. D. May A. M. Saffer B. Y. (2016). Suicide, suicide attempts, and suicidal ideation. Annu. Rev. Clin. Psychol. 12, 307–330. 10.1146/annurev-clinpsy-021815-093204 26772209

[B36] Kodama M. Fujioka T. Duman R. S. (2004). Chronic olanzapine or fluoxetine administration increases cell proliferation in hippocampus and prefrontal cortex of adult rat. Biol. Psychiatry 56 (8), 570–580. 10.1016/j.biopsych.2004.07.008 15476686

[B92] Koolhaas J. M. Coppens C. M. de Boer S. F. Buwalda B. Meerlo P. Timmermans P. J. (2013). The resident-intruder paradigm: A standardized test for aggression, violence and social stress. JoVE (77), e4367. 10.3791/4367 23852258 PMC3731199

[B37] Kraeuter A.-K. Guest P. C. Sarnyai Z. (2019a). The open field test for measuring locomotor activity and anxiety-like behavior. Pre-clinical models. Springer, 99–103. 10.1007/978-1-4939-8994-2_9 30535687

[B38] Kraeuter A. K. Guest P. C. Sarnyai Z. (2019b). The open field test for measuring locomotor activity and anxiety-like behavior. Methods Mol. Biol. 1916, 99–103. Humana Press Inc. 10.1007/978-1-4939-8994-2_9 30535687

[B39] Kukuia K. Ameyaw E. O. Mante P. K. Adongo D. W. Woode E. (2012). Screening of central effects of the leaves of *Mallotus oppositifolius* (Geiseler) Mull. Arg. in mice. Pharmacologia 3, 683–692. 10.5567/pharmacologia.2012.683.692

[B40] Kukuia K. K. Mante P. K. Woode E. Ameyaw E. O. Adongo D. W. (2014). Antidepressant effects of *Mallotus oppositifolius* in acute murine models. ISRN Pharmacol. 2014, 324063. 10.1155/2014/324063 25045543 PMC3972934

[B41] Kukuia K. K. E. Ameyaw E. O. Woode E. Mante P. K. Adongo D. W. (2016a). Enhancement of inhibitory neurotransmission and inhibition of excitatory mechanisms underlie the anticonvulsant effects of *Mallotus oppositifolius* . J. Pharm. Bioallied Sci. 8 (3), 253–261. 10.4103/0975-7406.183226 27413356 PMC4929967

[B42] Kukuia K. K. E. Ameyaw E. O. Woode E. Mante P. K. Adongo D. W. (2016b). Scientific evidence of plant with a rapid-onset and sustained antidepressant effect in a chronic model of depression: *Mallotus oppositifolius* . J. Basic Clin. Physiol. Pharmacol. 27 (5), 523–532. 10.1515/jbcpp-2015-0029 27089412

[B43] Kukuia K. K. E. Torbi J. Amoateng P. Adutwum-Ofosu K. K. Koomson A. E. Appiah F. (2022). Gestational iron supplementation reverses depressive-like behavior in post-partum Sprague Dawley rats: Evidence from behavioral and neurohistological studies. IBRO Neurosci. Rep. 12, 280–296. 10.1016/j.ibneur.2022.04.004 35746978 PMC9210498

[B88] Kulikov A. V. Osipova D. V. Naumenko V. S. Terenina E. Mormède P. Popova N. K. (2012). A pharmacological evidence of positive association between mouse intermale aggression and brain serotonin metabolism. Behav. Brain Res. 233 (1), 113–119. 10.1016/j.bbr.2012.04.031 22561036

[B44] Kumar A. Bansal D. Bajaj K. Sharma S. Srivastava V. (2003). Synthesis of some newer derivatives of 2-amino benzoic acid as potent anti-inflammatory and analgesic agents. Bioorg. Med. Chem. 11 (23), 5281–5291. 10.1016/s0968-0896(03)00529-7 14604692

[B46] Li L. Nandi I. Kim K. H. (2002). Development of an ethyl laurate-based microemulsion for rapid-onset intranasal delivery of diazepam. Int. J. Pharm. 237 (1-2), 77–85. 10.1016/s0378-5173(02)00029-7 11955806

[B47] Liebowitz M. R. Salman E. Nicolini H. Rosenthal N. Hanover R. Monti L. (2014). Effect of an acute intranasal aerosol dose of PH94B on social and performance anxiety in women with social anxiety disorder. Am. J. Psychiatry 171 (6), 675–682. 10.1176/appi.ajp.2014.12101342 24700254

[B48] Locci A. Pinna G. (2019). Social isolation as a promising animal model of PTSD comorbid suicide: Neurosteroids and cannabinoids as possible treatment options. Prog. Neuropsychopharmacol. Biol. Psychiatry 92, 243–259. 10.1016/j.pnpbp.2018.12.014 30586627

[B49] Luscher B. Shen Q. Sahir N. (2011). The GABAergic deficit hypothesis of major depressive disorder. Mol. Psychiatry 16 (4), 383–406. 10.1038/mp.2010.120 21079608 PMC3412149

[B51] Manchia M. Carpiniello B. Valtorta F. Comai S. (2017). Serotonin dysfunction, aggressive behavior, and mental illness: Exploring the link using a dimensional approach. ACS Chem. Neurosci. 8 (5), 961–972. 10.1021/acschemneuro.6b00427 28378993

[B52] Mann J. J. Metts A. V. Ogden R. T. Mathis C. A. Rubin-Falcone H. Gong Z. (2019). Quantification of 5-HT 1A and 5-HT 2A receptor binding in depressed suicide attempters and non-attempters. Arch. Suicide Res. 23 (1), 122–133. 10.1080/13811118.2017.1417185 29281590

[B53] Mann J. J. (2013). The serotonergic system in mood disorders and suicidal behaviour. Philos. Trans. R. Soc. Lond. B Biol. Sci. 368 (1615), 20120537. 10.1098/rstb.2012.0537 23440471 PMC3638390

[B90] McLafferty F. W. (2000). Wiley Registry of Mass Spectral Data. 6th Edn. New York, NY: Wiley.

[B54] National Research Council (1996). Guide for the care and use of laboratory animals. Institute of laboratory animal resources, commission on life Sciences. Washington, DC: National Academy of Sciences.

[B55] Nwaehujor C. O. Ezeja M. I. Udeh N. E. Okoye D. N. Udegbunam R. I. (2014). Anti-inflammatory and anti-oxidant activities of Mallotus oppositifolius (Geisel) methanol leaf extracts. Arabian J. Chem. 7 (5), 805–810. 10.1016/j.arabjc.2012.03.014

[B56] Ohira K. Takeuchi R. Shoji H. Miyakawa T. (2013). Fluoxetine-induced cortical adult neurogenesis. Neuropsychopharmacology 38 (6), 909–920. 10.1038/npp.2013.2 23303069 PMC3629401

[B57] Ozcan A. S. (2017). Filopodia: A rapid structural plasticity substrate for fast learning. Front. Synaptic Neurosci. 9, 12. 10.3389/fnsyn.2017.00012 28676753 PMC5476769

[B58] Popova N. K. Naumenko V. S. (2019). Neuronal and behavioral plasticity: The role of serotonin and BDNF systems tandem. Expert Opin. Ther. Targets 23 (3), 227–239. 10.1080/14728222.2019.1572747 30661441

[B59] Porsolt R. D. Brossard G. Hautbois C. Roux S. (2001). “Rodent models of depression: Forced swimming and tail suspension behavioral despair tests in rats and mice,” in Current protocols in neuroscience/editorial board. Editor Jacqueline N. (Crawley. [et al.] Chapter 8. 10.1002/0471142301.ns0810as14 18428536

[B60] Quadros I. M. Takahashi A. Miczek K. A. (2020). “Serotonin and aggression—An update,”. Handbook of behavioral neuroscience (Elsevier B.V.), 31, 635–663. 10.1016/B978-0-444-64125-0.00037-2

[B63] Risher W. C. Ustunkaya T. Singh Alvarado J. Eroglu C. (2014). Rapid Golgi analysis method for efficient and unbiased classification of dendritic spines. PLoS One 9 (9), e107591. 10.1371/journal.pone.0107591 25208214 PMC4160288

[B64] Rosa P. B. Bettio L. E. B. Neis V. B. Moretti M. Kaufmann F. N. Tavares M. K. (2021). Antidepressant-like effect of guanosine involves activation of AMPA receptor and BDNF/TrkB signaling. Purinergic Signal. 17 (2), 285–301. 10.1007/s11302-021-09779-6 33712981 PMC8155134

[B65] Saeed N. M. El-Demerdash E. Abdel-Rahman H. M. Algandaby M. M. Al-Abbasi F. A. Abdel-Naim A. B. (2012). Anti-inflammatory activity of methyl palmitate and ethyl palmitate in different experimental rat models. Toxicol. Appl. Pharmacol. 264 (1), 84–93. 10.1016/j.taap.2012.07.020 22842335

[B67] Sherif A. T. Y. (2021). *In vitro* evaluation of purple inflorescence of Gomphrena globosa (L.) extracts for anti-inflammatory activity and its GC/MS profile. Asian J. Biol. Life Sci. 10 (1), 123–131. 10.5530/ajbls.2021.10.19

[B68] Siegel A. Victoroff J. (2009). Understanding human aggression: New insights from neuroscience. Int. J. Law Psychiatry 32 (4), 209–215. 10.1016/j.ijlp.2009.06.001 19596153

[B71] Stein S. (1990). National institute of standards and technology (NIST) mass spectral database and software, Version.

[B72] Steru L. Chermat R. Thierry B. Simon P. (1985). The tail suspension test: A new method for screening antidepressants in mice. Psychopharmacology 85 (3), 367–370. 10.1007/BF00428203 3923523

[B73] Stone M. Laughren T. Jones M. L. Levenson M. Holland P. C. Hughes A. (2009). Risk of suicidality in clinical trials of antidepressants in adults: Analysis of proprietary data submitted to US Food and Drug Administration. BMJ (Online) 339 (7718), 339. 10.1136/bmj.b2880 PMC2725270 19671933

[B74] Stübner S. Grohmann R. Greil W. Zhang X. Müller-Oerlinghausen B. Bleich S. (2018). Suicidal ideation and suicidal behavior as rare adverse events of antidepressant medication: Current report from the AMSP multicenter drug safety surveillance project. Int. J. Neuropsychopharmacol. 21 (9), 814–821. 10.1093/ijnp/pyy048 29939264 PMC6119288

[B75] Studer E. Näslund J. Andersson E. Nilsson S. Westberg L. Eriksson E. (2015). Serotonin depletion-induced maladaptive aggression requires the presence of androgens. PLoS ONE 10 (5), e0126462. 10.1371/journal.pone.0126462 25978464 PMC4433101

[B77] Underwood M. D. Kassir S. A. Bakalian M. J. Galfalvy H. Mann J. J. Arango V. (2012). Neuron density and serotonin receptor binding in prefrontal cortex in suicide. Int. J. Neuropsychopharmacol. 15 (4), 435–447. 10.1017/S1461145711000691 21733245 PMC4167642

[B78] van Heeringen C. Bijttebier S. Godfrin K. (2011). Suicidal brains: A review of functional and structural brain studies in association with suicidal behaviour. Neurosci. Biobehav. Rev. 35 (3), 688–698. 10.1016/j.neubiorev.2010.08.007 20826179

[B91] Walz J. C. Stertz L. Fijtman A. dos Santos B. T. de Almeida R. M. M. (2013). Tryptophan diet reduces aggressive behavior in male mice. Psychol. Neurosci. 6, 397–401.

[B79] Wang Y. Wang X. Chen J. Li S. Zhai H. Wang Z. (2019). Melatonin pretreatment attenuates acute methamphetamine-induced aggression in male ICR mice. Brain Res. 1715, 196–202. 10.1016/j.brainres.2019.04.002 30953606

[B80] World Health Organization (2017). Depression and other common mental disorders: Global health estimates.

[B81] World Health Organization (2018). Suicide data. 2017. [WebCite Cache ID 6tHDxzbVs] URL: http://www.who.int/mental_health/prevention/suicide/suicideprevent/en/09 06, accessed 2017.

[B82] Yanowitch R. Coccaro E. F. (2011). The neurochemistry of human aggression. Adv. Genet. 75, 151–169. Academic Press Inc. 10.1016/B978-0-12-380858-5.00005-8 22078480

[B83] Ye S. Zhong J. Huang J. Chen L. Yi L. Li X. (2021). Protective effect of plastrum testudinis extract on dopaminergic neurons in a Parkinson's disease model through DNMT1 nuclear translocation and SNCA's methylation. J. Biomed. Pharmacother. 141, 111832. 10.1016/j.biopha.2021.111832 34153844

[B84] Zhu X.-L. Chen J.-J. Han F. Pan C. Zhuang T.-T. Cai Y.-F. (2018). Novel antidepressant effects of Paeonol alleviate neuronal injury with concomitant alterations in BDNF, Rac1 and RhoA levels in chronic unpredictable mild stress rats. Psychopharmacology 235 (7), 2177–2191. 10.1007/s00213-018-4915-7 29752492

[B85] Zubrick S. R. Hafekost J. Johnson S. E. Sawyer M. G. Patton G. Lawrence D. (2017). The continuity and duration of depression and its relationship to non-suicidal self-harm and suicidal ideation and behavior in adolescents 12–17. J. Affect. Disord. 220, 49–56. 10.1016/j.jad.2017.05.050 28595098

